# Silencing *amorpha-4,11-diene synthase* Genes in *Artemisia annua* Leads to FPP Accumulation

**DOI:** 10.3389/fpls.2018.00547

**Published:** 2018-05-29

**Authors:** Theresa M. Catania, Caroline A. Branigan, Natalia Stawniak, Jennifer Hodson, David Harvey, Tony R. Larson, Tomasz Czechowski, Ian A. Graham

**Affiliations:** Centre for Novel Agricultural Products, Department of Biology, University of York, York, United Kingdom

**Keywords:** *Artemisia annua*, artemisinin, sesquiterpene, glandular trichome, amorpha-4,11-diene synthase, farnesyl pyrophosphate

## Abstract

*Artemisia annua* is established as an efficient crop for the production of the anti-malarial compound artemisinin, a sesquiterpene lactone synthesized and stored in Glandular Secretory Trichomes (GSTs) located on the leaves and inflorescences. *Amorpha-4,11-diene* synthase (AMS) catalyzes the conversion of farnesyl pyrophosphate (FPP) to amorpha-4,11-diene and diphosphate, which is the first committed step in the synthesis of artemisinin. FPP is the precursor for sesquiterpene and sterol biosynthesis in the plant. This work aimed to investigate the effect of blocking the synthesis of artemisinin in the GSTs of a high artemisinin yielding line, Artemis, by down regulating *AMS*. We determined that there are up to 12 *AMS* gene copies in Artemis, all expressed in GSTs. We used sequence homology to design an RNAi construct under the control of a GST specific promoter that was predicted to be effective against all 12 of these genes. Stable transformation of Artemis with this construct resulted in over 95% reduction in the content of artemisinin and related products, and a significant increase in the FPP pool. The Artemis *AMS* silenced lines showed no morphological alterations, and metabolomic and gene expression analysis did not detect any changes in the levels of other major sesquiterpene compounds or sesquiterpene synthase genes in leaf material. FPP also acts as a precursor for squalene and sterol biosynthesis but levels of these compounds were also not altered in the *AMS* silenced lines. Four unknown oxygenated sesquiterpenes were produced in these lines, but at extremely low levels compared to Artemis non-transformed controls (NTC). This study finds that engineering *A. annua* GSTs in an Artemis background results in endogenous terpenes related to artemisinin being depleted with the precursor FPP actually accumulating rather than being utilized by other endogenous enzymes. The challenge now is to establish if this precursor pool can act as substrate for production of alternative sesquiterpenes in *A. annua*.

## Introduction

*Artemisia annua (A. annua)* is a herbaceous plant from the Asteraceae family native to Asia, known to synthesize the leading antimalarial compound artemisinin a sesquiterpene lactone, within its glandular secretory trichomes (GSTs) (Duke et al., 1994; Olsson et al., 2009). The GSTs of *A. annua* are biseriate glandular trichomes made up of 10 cells topped with a secretory sac. The secretory sac is bounded by a cuticle proximal to the three apical pairs of cells. This arrangement allows phytotoxic compounds such as artemisinin to be sequestered away from the plant, preventing autotoxicity (Duke and Paul, 1993; Duke et al., 1994; Ferreira and Janick, 1995). The distribution of *A. annua* GSTs across leaves, stems, and inflorescences, combined with the relative ease of artemisinin extraction using organic solvents, has made feasible the commercial scale growth and processing of whole plants for production of the compound as an active pharmaceutical ingredient in Artemisinin Combination Therapies (ACTs). The efficiency of the *A. annua* production system, which can yield artemisinin at greater than 1% lead dry weight and 41.3 Kg per Ha (Ferreira et al., 2005) has meant that it persists as the most efficient and economically feasible platform for production of artemisinin today. With *A. annua* as the sole source of artemisinin, demands for the drug have influenced farmers on whether to grow the crop or not, leading to large market price fluctuations (highs of $1,100 in 2005 to less than $250 per kilogram in 2007 and again in 2015 (Van Noorden, 2010; Peplow, 2016). A desire to both stabilize and reduce costs in the supply chain has driven research into yield improvement through modern marker assisted plant breeding and genetic engineering methods and through engineering artemisinin (or precursor) synthesis in heterologous hosts (Ferreira et al., 2005; Han et al., 2006; Graham et al., 2010; Zhang et al., 2011; Paddon and Keasling, 2014; Tang et al., 2014; Pulice et al., 2016).

The commercial importance of artemisinin synthesis has stimulated ongoing research into the biosynthetic pathways and metabolic capabilities of *A. annua* GSTs. Transcriptomic analysis of GSTs from *A. annua* has identified multiple genes including cytochrome P450s and terpene synthases, which have been subsequently characterized in detail and their trichome-specific expression patterns confirmed (Olsson et al., 2009; Wang et al., 2009; Graham et al., 2010; Olofsson et al., 2011, 2012; Soetaert et al., 2013). Metabolomic analysis in *A. annua* has identified almost 600 secondary and/or specialized metabolites, whose production can be linked to the expression of the identified synthases (Brown, 2010). This suggests that the GSTs of *A. annua* are highly evolved terpenoid-producing factories, with the potential for producing and storing a diverse range of compounds.

**Figure 1:**
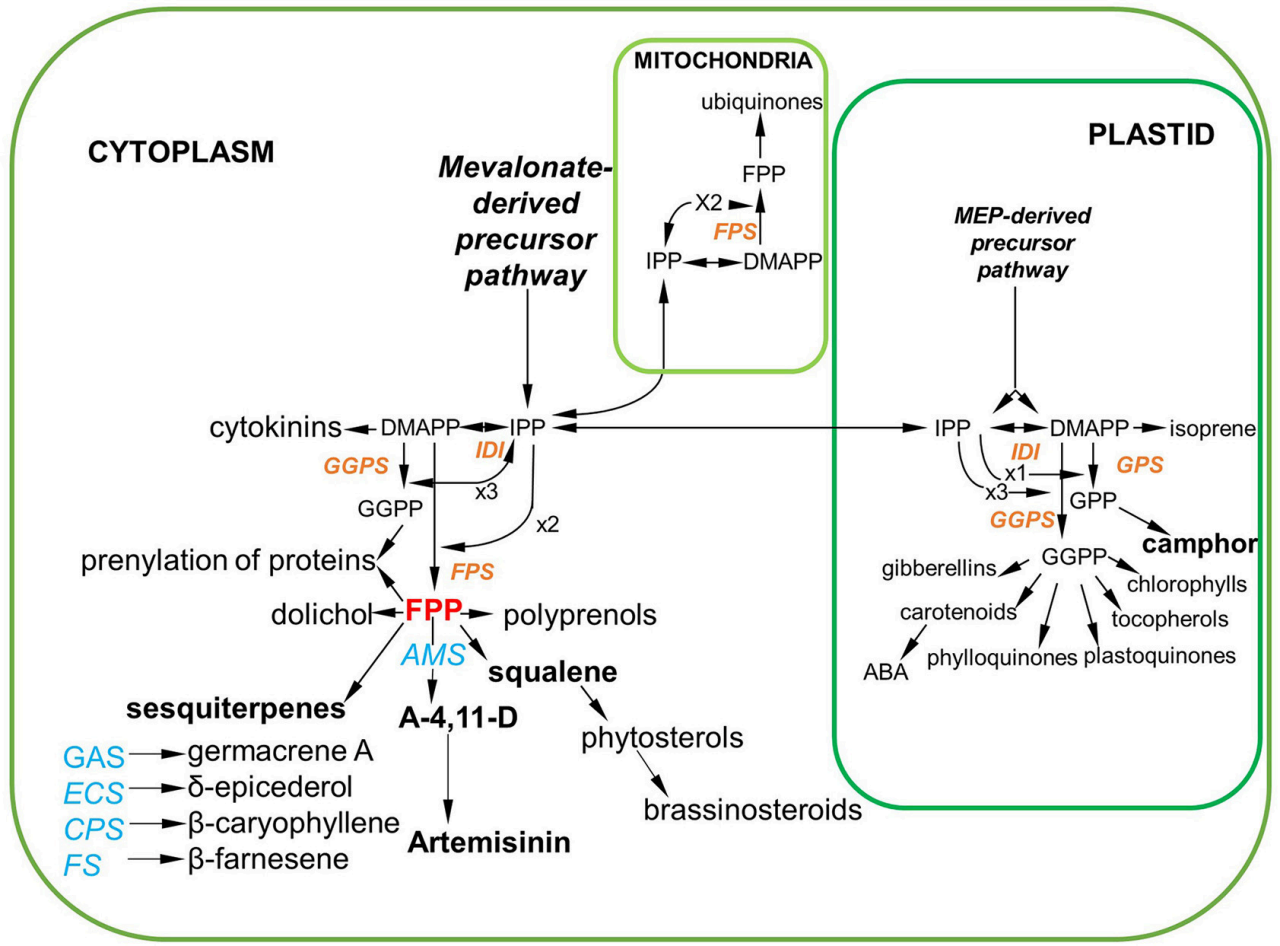
Summary of sesquiterpene production in *Artemisia annua* via the mevalonate (MVA) pathway in the cytosol FPP is highlighted as the key precursor not only for artemisinin synthesis but also for Squalene and other sequiterpenes. The plastidial methyl erythritol phosphate (MEP) pathway that leads to monoterpene production is also included. enzymes in red have been cloned from *A. annua* and the sesquiterpene synthases and squalene synthases also cloned from *A. annua* are in blue (DMAPP, dimethylallyl diphosphate; GGPP, geranyl geranyl diphosphate; GPP, geranyl pyrophosphate; GGPS, geranylgeranyl pyrophosphate synthase; IPP, isopentenyl diphosphate; IDI, isopentyl diphosphate isomerase; ABA, abscisic acid; *FPS, farnesyl diphosphate synthase*; FPP, farnesyl pyrophosphate; *AMS, amorpha-4,11-diene synthase; SQS, squalene synthase; GAS, germacrene A synthase; ECS, δ-epicederol synthase; CPS, caryophyllene synthase; FS, beta farnesene synthase)*.

The biosynthesis of terpenoids including artemisinin in *A. annua* starts with the biosynthetic precursors, isopentenyl diphosphate (IPP) and its isomer dimethylallyl diphosphate (DMAPP), which are in turn products of the methyl erythritol phosphate (MEP) and mevalonate (MVA) pathways (Croteau et al., 2000; Weathers et al., 2006; Wu et al., 2006). IPP and DMAPP precursors for the synthesis of farnesyl pyrophosphate (FPP), which is in turn the immediate precursor of both sterols and sesquiterpenes including artemisinin (Figure 1). Currently 5 sesquiterpene synthases have been cloned from *A. annua, amorpha-4,11-diene synthase* (*AMS*), the first step in artemisinin synthesis (Bouwmeester and Wallaart, 1999); *caryophyllene synthase* (*CPS*) (Cai et al., 2002); *germacrene A synthase* (*GAS*) (Bertea et al., 2006); δ*-epicederol synthase* (*ECS*) (Mercke et al., 1999) and *beta farnesene synthase* (*FS*) (Picaud et al., 2005). The expression of these synthases is shown to be predominantly in GSTs and young leaf tissue (Graham et al., 2010; Olofsson et al., 2011) Other sesquiterpenes such as guaianes, longipinanes, and eudesmanes have also been isolated suggesting the expression of other sesquiterpene synthases (Brown, 2010; Olofsson et al., 2011). These synthases all compete for the precursor FPP, and engineering of the pathway from this point by overexpression of *AMS* (Ma et al., 2009, 2015; Han et al., 2016) or silencing of CPS, BFS, GAS, and ECS (Chen et al., 2011; Lv et al., 2016) has been a strategy for increasing artemisinin production. FPP is also utilized by *squalene synthase* (*SQS*) in the first committed step to sterol synthesis. Silencing of this synthase is shown to remove the sink on FPP from squalene and sterol production resulting in increased artemisinin yield (Yang et al., 2008; Zhang et al., 2009). In these studies flux is altered in the artemisinin pathway leading to increased artemisinin yields when compared to wild type, Ma et al. (2015) and Lv et al. (2016) also show that by manipulating the pathway other endogenous terpenes were also affected.

Czechowski et al. (2016) showed a mutation disrupting the amorpha-4,11-diene C-12 oxidase (CYP71AV1) in the high artemisinin yielding cultivar Artemis, produced a novel sesquiterpene epoxide derivative at levels similar to artemisinin (arteannuin X; 0.3–0.5% of leaf dry weight). This discovery demonstrates the possibility of engineering *A. annua* GSTs to produce alternative, potentially useful, sesquiterpenes at commercially viable levels. GSTs are targeted for engineering based on their ability to synthesize and store specialized metabolites (Huchelmann et al., 2017). Efforts to engineer GSTs reported in the literature include examples in tobacco, and tomato. Tissier et al. (2012) engineered tobacco GSTs to successfully produce casbene and taxadiene, although production was at levels lower than that for endogenous diterpenoids. The engineering of tomato trichomes has also been carried out by expressing sesquiterpene synthases from wild relatives to confer pest resistance (Bleeker et al., 2012; Yu and Pichersky, 2014). Kortbeek et al. (2016) have also used the GSTs of tomato as a platform for engineering sesquiterpenoid production by the overexpression of an avian FPS (farnesyl diphosphate synthase), to increase FPP availability for sesquiterpene production. Engineering of *A. annua* trichomes has been mainly centered on enhancing artemisinin production by constitutively expressing upstream enzymes, artemisinin biosynthetic genes and transcription factors (Tang et al., 2014; Xie et al., 2016; Ikram and Simonsen, 2017). More complex pathway regulation has also been attempted—a patent by Tang et al. (2011) describes a method for using the trichome specific *cyp71av1* promoter to drive both the expression of an *ADS (amorpha-4,11- diene synthase /AMS)* silencing construct and the patchouli alcohol biosynthesis enzyme, to allow the production of patchouli alcohol in *A. annua*.

The objective of the current work was to silence AMS in the GSTs of Artemis a high artemisinin yielding cultivar. Silencing of AMS in this background allowed us to investigate how carbon flux through FPP is affected, in contrast to previous studies performed in low-ART yielding systems where alternative products accumulate (Ma et al., 2015; Lv et al., 2016). We demonstrate that removing artemisinin and related compounds elevates the FPP pool with only minor increases in alternative endogenous metabolites. We conclude that such manipulations, done in a carefully selected genetic background, have the potential to provide a clean chemical background for pathway engineering, without detrimental effects on plant growth and development.

## Methods

### Plant material

The *A. annua* cultivar Artemis, an F1 hybrid from Mediplant (Conthey, Switzerland) (described in Graham et al., 2010) was used to generate stably transformed material.

### AMS gene copy determination by qPCR

DNA extraction was carried out on 30–50 mg of fresh leaf material harvested from plants growing in the glasshouse and prepared following the methods as described in Graham et al. (2010) and Czechowski et al. (2016).

Three technical replications of a 10 μl reaction containing 1 ng of leaf genomic DNA from single plants, 200 nM gene-specific primers in 1x Power SYBER Green PCR Master Mix (Life Technologies Ltd.), were run on a ViiA7 Real-Time PCR system (Life Technologies Ltd.) The gene specific primers were as follows:

*AMS*_3′endF: TCTACTCGTTTATCCTATGAGTATATGACT ACC
*AMS*_3′endR: GGCTATGCACGAAGGATTGGT
*AMS*_5′endF: TTACCGAAATACAACGGGCAC
*AMS*_5′endR: TTGGCAACCTTTTCCAAAGG

Amplification conditions and data normalization were as described in Czechowski et al. (2016).

### RNA isolation and cDNA synthesis

Fresh young leaf material (leaves 1–5) (30–50 mg) from 12-week-old glasshouse grown cuttings was harvested and flash frozen in liquid nitrogen for RNA extraction. The extraction was carried out using the Qiagen RNAeasy kit following the manufacturer's plant protocol including the on column Qiagen DNase treatment. Extracted RNA was quantified spectrophotometrically using the NanoDrop-8000 (NanoDrop products). cDNA was synthesized from 3 μg of the extracted RNA using Invitrogen superscript II reverse transcriptase kit (Thermo Fischer Scientific) using the oligo (dT) primer following the manufacturers protocol.

### Construction of hpRNA vector targeting the AMS gene

Two sections of the *AMS* gene (AF138959) from bases 96–192 and 1485–1615 were selected and joined to create a 227 bp sequence which was checked for its specificity to the *AMS* target relative to other sesquiterpene synthases from *A. annua* (Mercke et al., 1999; Cai et al., 2002; Picaud et al., 2005; Bertea et al., 2006; Supplemental Figure 1). This sequence was then placed in a forward and reverse direction either side of the Chalcone synthase A intron (petunia hybrida) to create a hairpin construct (Watson et al., 2005), this was driven by the trichome specific promoter *cypav171* (Wang et al., 2011). The full construct was synthesized by GENEART Thermo life technologies. The 3.8 kb construct was cloned into the pRSC2 binary vector and transformed into stratagene solopack gold competent cells. The resulting colonies were tested by PCR using Promega Gotaq and primers designed for the pRSC2 vector.

AP1435 pRSC2_activ TAACATCCAACGTCGCTTTCAG
AP1436 pRSC2_RB_in GCCAATATATCCTGTCAAACAC

Positive colonies were confirmed by sequencing and the binary vector was then transferred into *Agrobacterium tumefaciens* (LBA4404) by electroporation and 100 μl glycerol stocks set up for subsequent transformations. Forty eight hours prior to transformation the agrobacterium pre-cultures were set up from glycerol stocks in Luria-Bertani broth (LB) including antibiotic selection (50 mg/L rifampicin, spectomycin and streptomycin). After 24 h, a 50 ml main culture was set up and allowed to grow overnight to an optical density (OD) of between 0.3 and 0.8. At this stage the cultures were spun down and resuspended in co-cultivation media (Murashige and Skoog medium (MS) with 3% sucrose and 100 uM acetosyringone) to an OD of 0.2. The culture was then left to shake at 28 °C for 2 h.

### *Artemisia annua* transformation

Artemis seed were surface sterilized for 1 h using chlorine vapor [3% HCL in water + one presept tablet (Advanced sterilization products)] in a sealed box. Seeds were sown into sterile glass jars on MS basal media containing 3% sucrose, 1x MS vitamins and 0.8% plant agar. After sowing the jars were closed and sealed with parafilm and transferred to a growth room 16 h daylength at 29°C to germinate and grow for 2.5 weeks.

The first true leaves of 2.5-week-old seedlings were excised and immersed in petri dishes into either an agrobacterium suspension, or for non-transformed controls (NTC), co-cultivation media without agrobacterium, and placed on a rotary platform. After 15 min, the explants were blotted on sterile filter paper and transferred to labeled co-cultivation plates (MS with 3% sucrose and 100 *u*M acetosyringone +0.8% plant agar) the plates were wrapped in foil and stored in the growth room at 25°C. After 48 h the explants were transferred to selection plates (MS medium with 3% sucrose, 0.5 mg/L 6-benzylaminopurine (BAP) and 0.05 mg/L α-naphthalene acetic acid (NAA), 0.8% agar, 500 mg/L carbenicillin and 15 mg/L kanamycin. NTC explants were plated out without kanamycin selection. Explants were transferred to fresh plates after a week and thereafter every 2 weeks. Shoots were excised from the plates as they emerged and placed onto shooting medium in jars (MS medium with 3% sucrose, 0.5 mg/L BAP, and 0.05 mg/L NAA, 0.8% plant agar, 500 mg/L carbenicillin, 15 mg/L kanamycin), and transferred on every 3 weeks. NTC shoots were placed into shooting medium containing no antibiotic. Once the shoots were well-established they were transferred to rooting medium (1/2 MS medium, 1% sucrose, 0.6% plant agar, 500 mg/L carb, 15 mg/L kanamycin, (NTCs with no antibiotics). Once roots had begun to appear, the shoots were transferred to F2+S compost in P40s and kept in green propagator trays with lids on to maintain humidity. Once well rooted the plants were hardened off and transferred to 4-inch pots in F2 compost and grown in glasshouse facilities at the University of York under long day conditions maintained with supplemental lighting and temperature between 22 and 25°C. Putatively transformed lines were confirmed by PCR for the presence of the NPTII gene using the Phire plant direct PCR kit (Thermo Fisher Scientific). Transformed plants alongside NTCs were propagated via cuttings and grown in triplicate for 12 weeks to provide material for DNA and RNA extractions and for metabolite profiling by UPLC-MS and GC-MS.

### Quantitative RT-PCR

Expression levels of *amorpha-4,11-diene synthase* (AMS), squalene synthase (SQS), germacrene A synthase (GAS); δ-epicederol synthase (ECS); beta farnesene synthase (BFS); and caryophyllene synthase (CPS) relative to ubiquitin (UBI; Genbank accession: GQ901904) were determined by quantitative RT-PCR. Expression levels of each gene were determined for cDNA from NTC and transformed young leaf material prepared as described above (section AMS Gene Copy Determination by qPCR). Gene-specific primers used were:

*AMS*_For     5′- GGGAGATCAGTTTCTCATCTATGAA- 3′
*AMS*_Rev     5′- CTTTTAGTAGTTGCCGCACTTCTT-3′
*CPS*_For     5′-CAACGATGTAGAAGGCTTGCTTGA-3′
*CPS*_Rev     5′-GTAGATAGTGTTGGGTTGGTGTGA-3′
*ECS*_For     5′-GCAACAAGCCTACGAATCACTCAA-3′
*ECS*_Rev     5′-CGTGAAAAATTAAGGACCCTCATAG-3′
*GAS*_For     5′-CTCGTTACTCCTTGGCAAGAATCAT-3′
*GAS*_Rev     5′-GCTCCATAGCACTAATATCCCACTT-3′
*SQS*_For     5′-GACCAGTTCCACCATGTTTCTACT-3′
*SQS*_Rev     5′-GCTTTGACAACCCTATTCCAACAAG-3′
*FS*_For       5′-GCAAAAGAGTTGGTTCGCAATTAC-3′
*FS*_Rev       5′-GTACCCCTCTTTTAGCCATCTGG-3′
*UBI*_For     5′-TGATTGGCGTCGTCTTCGA-3′
*UBI*_Rev     5′-CCCATCCTCCATTTCTAGCTCAT-3′

Amplification conditions and data analysis were as described in Graham et al. (2010) and Czechowski et al. (2016).

### Metabolite analysis by UPLC-MS and GC-MS

Three replicate cuttings from the NTC and each transformed line were grown in 4-inch pots under 16-h days for 12 wk. Metabolite profiles were generated from 50 mg fresh weight pooled samples of leaves at young (first emerging leaf to leaf 6) or mature (the tips of leaves 11–13) developmental stages the fresh leaf samples collected were stored at −80°C. Dry leaf material was obtained from 18-week-old plants, cut just above the zone of senescing leaves, and dried for 14 d at 40°C. Leaves were stripped from the plants, and leaf material was sieved through 5-mm mesh to remove small stems. Trichome-specific metabolites (Supplemental Figure 2) were extracted and analyzed as previously described (Graham et al., 2010; Czechowski et al., 2016).

### Architecture and leaf traits and trichome density

Height, leaf area and trichome density were also measured on the NTC and transformed lines as described in Graham et al. (2010).

### FPP quantification

FPP quantification was carried out on isolated GSTs and young leaf material using pooled leaf tips (meristem to leaf 6) collected from the apical meristem and each axillary branch counting down to the axillary branch at leaf position 20. Glandular trichomes were isolated as described in Graham et al. (2010). The young leaf material was ground under liquid nitrogen and 1 gram weighed out for extraction. Both the isolated trichomes and the ground leaf were extracted in methanol:water (7:3, v/v), including a total of 0.3 μg farnesyl S-thiolodiphosphate (FSPP; Echelon Biosciences) added as an internal standard. Extracts were processed according to Nagel et al. (2014). Briefly, each extract was passed through a Chromabond HX RA column (150 mg packing), which had first been conditioned with 5 ml methanol and 5 ml of water, and compounds eluted under gravity with 3 ml of 1 M ammonium formate in methanol. The eluate was evaporated under a stream of nitrogen to dryness, dissolved in 250 μL of water:methanol (1:1.v/v), and a 2 μL aliquot injected on a Waters Acquity I-Class UPLC system interfaced to a Thermo Orbitrap Fusion Tribrid mass spectrometer under Xcalibur 4.0 control. Compounds were eluted on a Waters Acquity C18 BEH column (2.1 mm × 100 mm, 1.7 μm) at 50°C using the following binary gradient program: solvent A = 20 mM ammonium bicarbonate + 0.1% triethylamine; solvent B = 4:1 acetonitrile:water + 0.1% triethylamine; flowrate 0.4 ml/min; 0–100% B linear gradient over 4 min. Post-column, compounds were ionized using a heated electrospray source (vaporizer = 358°C; N_2_ flows for sheath/aux/sweep = 45/13/1 arbitrary units; source = 2.5 kV; ion transfer tube = −30 V and 342°C; tube lens = −40 V). Data was acquired in full scan mode with the following settings: orbitrap resolution = 15 k, 100–500 m/z range, max ion time 100 ms, 1 microscan, AGC target = 200,000, S-Lens RF Level = 60. FPP eluted at ~2.4 min and the internal standard (FSPP) at ~2.5 min. The deprotonated pseudomolecular ions ([M-H]^−^) of 381.1227 and 397.0998 for FPP and FSPP, respectively, were used for quantification (±5 ppm window) against a 0.1–100 μM linear FPP/FSPP response ratio calibration curve (*R*^2^ = 0.99), using Xcalibur 4.0 software (Thermo). For less complex trichome-only samples, a Thermo LTQ Orbitrap Classic instrument was used in ion trap mode.

### Sterol quantification

A 200 mg sample of pooled leaf material from the NTC and AMS silenced lines was ground and extracted by sonication in dichloromethane as described by Zhang et al. (2009). Extracts were centrifuged, the upper phase collected and a 1 uL aliquot analyzed by GC-MS as described in Czechowski et al. (2016), except that the final GC oven temperature and hold time were increased to 350°C and 8 min, respectively, to ensure elution of sterols and squalene. ChomaTof 4.0 software (Leco) was used for spectral processing, to produce deconvoluted spectra for identification against the NIST 2014 database and authentic standards. ChromaTof-selected unique masses were used to generate and integrate peak areas under selected ion traces for quantification against authentic sterol and squalene standards.

### Data analysis

Peak lists for UPLC-MS and GC-MS data were obtained and processed using bespoke R scripts as described in Czechowski et al. (2016). Data from GC-MS and UPLC-MS for the young mature and dried leaf were analyzed by ANOVAs using GENSTAT software (VSN international) with the Bonferroni *post-hoc* test (*p* ≤ 0.05) to compare between NTC and transformed lines.

## Results

### Silencing the first committed step in artemisinin production results in accumulation of the sesquiterpene precursor FPP in glandular secretory trichomes of *A. annua*

**Figure 2:**
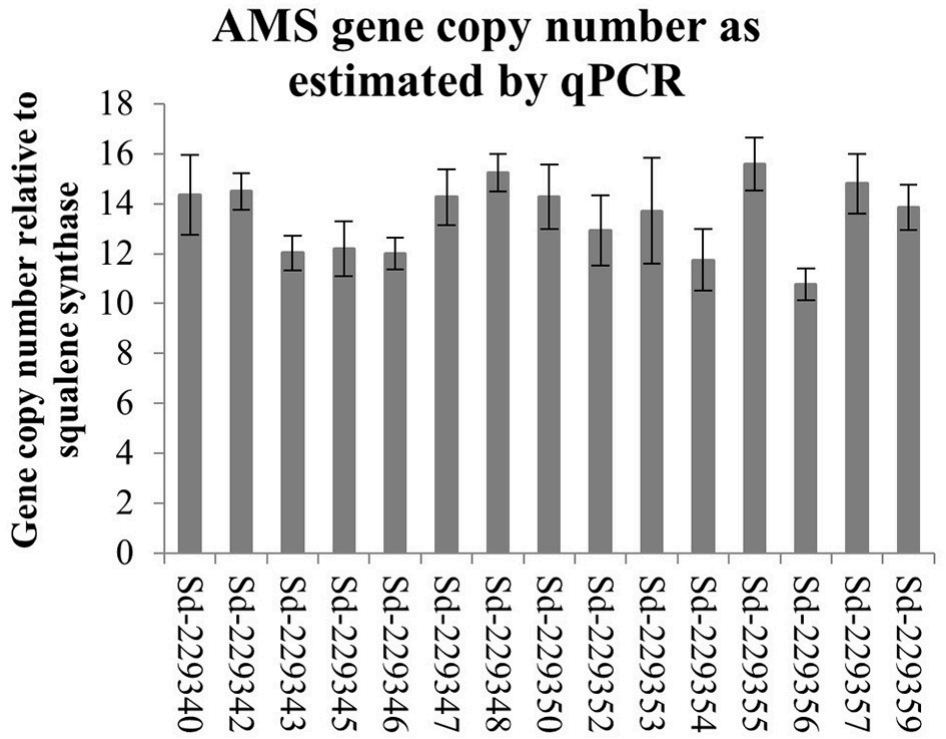
*AMS* gene copy number in 15 individual lines of the *A. annua* cultivar Artemis estimated by qPCR. Error bars represent standard error (*n* = 4). Sd prefixed numbers represent individual Artemis plants.

**Figure 3:**
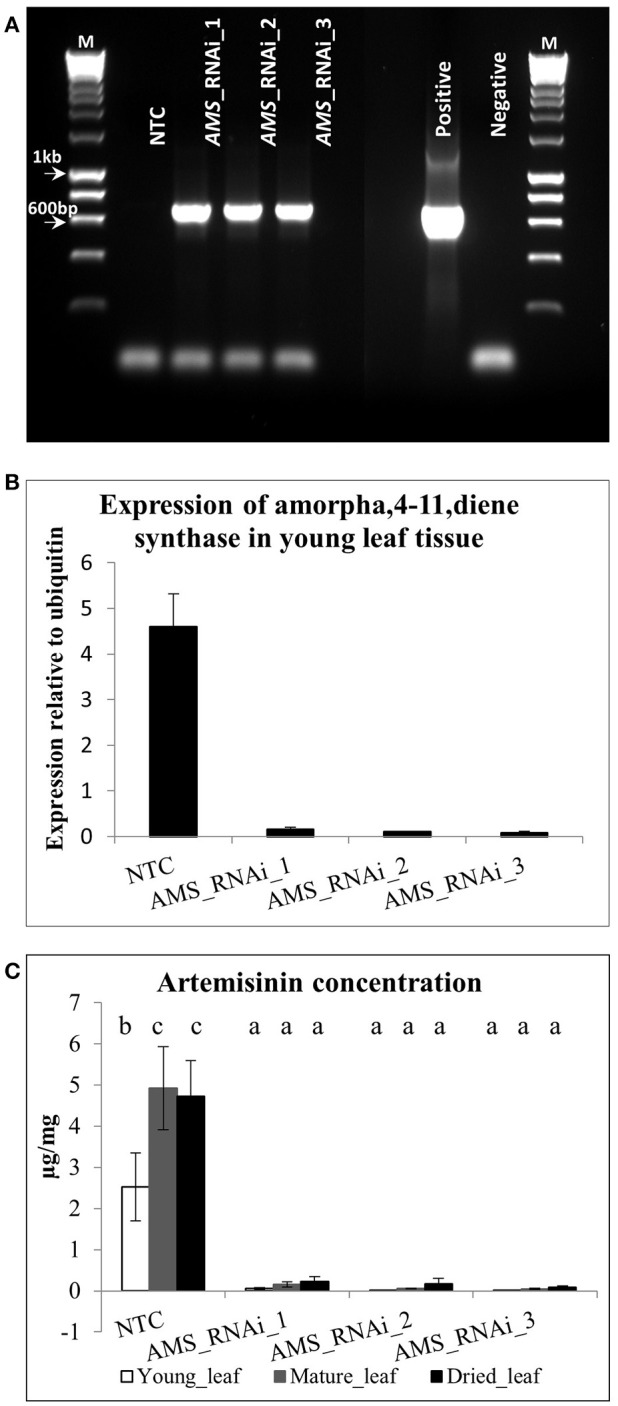
**(A)** PCR results for *NPTII* primers M − 1 kb hyperladder™ (Bioline), **(B)** Q-RT-PCR of AMS gene expression and **(C)** Artemisinin concentration (μg/mg extracted dry weight) in young and mature leaves and dried pooled leaf material for the NTC and *AMS* silenced lines. Error bars ±SD (NTC—*n* = 6, *AMS*_RNAi lines—*n* = 3). Letters represent Bonferroni test results after ANOVA, groups not sharing letters indicate statistically significant differences (*p* ≤ 0.05).

**Figure 4:**
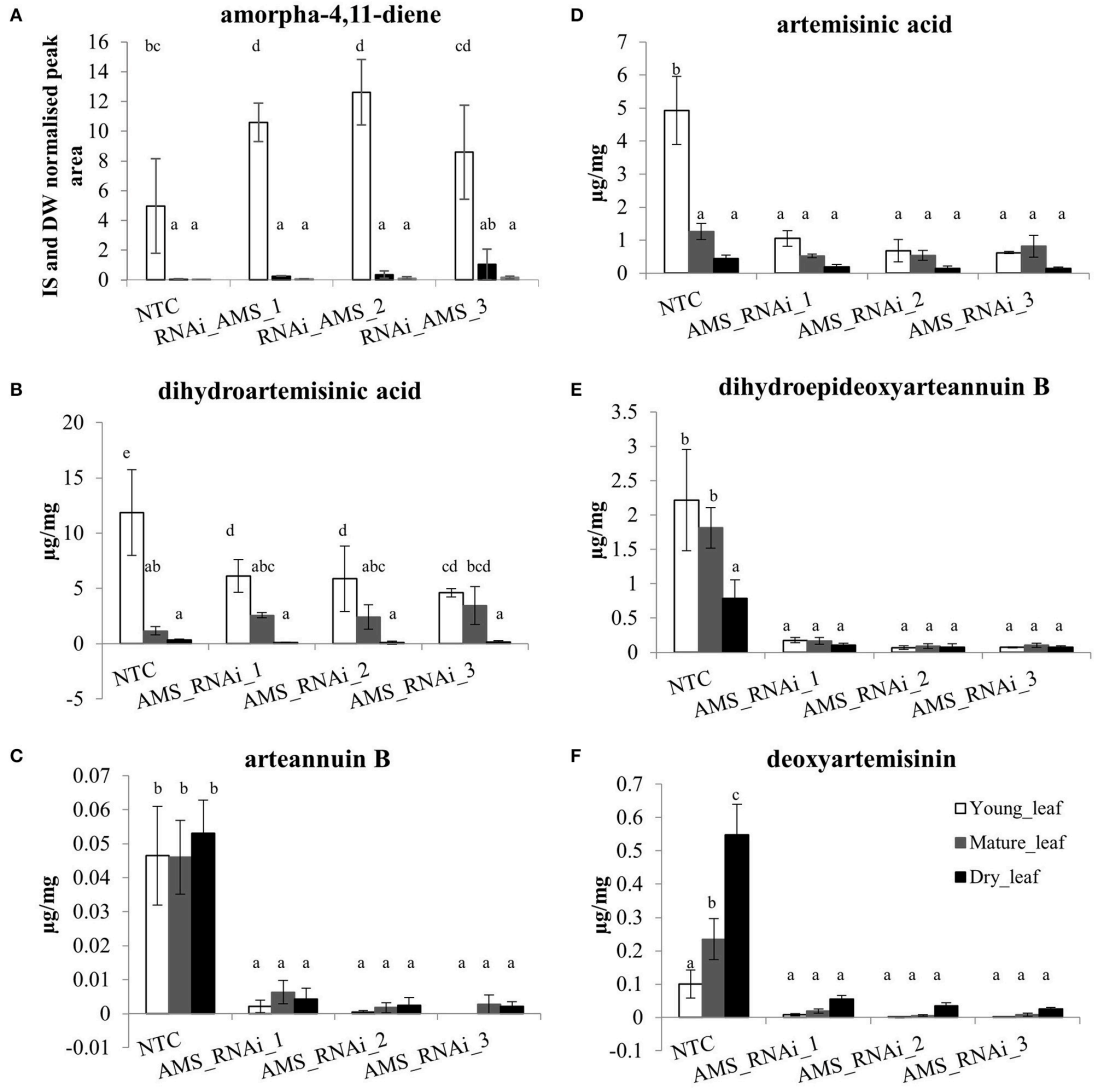
The concentration (μg/mg extracted dry weight) of known artemisinin pathway compounds in young leaves, mature leaves and dried pooled leaf material for NTC and *AMS*_RNAi lines. **(A)** amorpha-4,11-diene, **(B)** artemisinic acid, **(C)** dihydroartemisinic acid, **(D)** dihydroepideoxyarteannuin B, **(E)** arteannuin B, **(F)** deoxyartemisinin. Error bars ±SD (NTC—*n* = 6, *AMS*_RNAi lines—*n* = 3). Letters represent Bonferroni test results after ANOVA, groups not sharing letters indicate statistically significant differences (*p* ≤ 0.05).

The *Amorpha-4,11-diene synthase* (*AMS*) enzyme responsible for catalyzing the first committed step in artemisinin production is encoded by a small gene family averaging 12 copies in the Artemis F1 hybrid variety (Figure 2). We built a hairpin-based gene silencing construct that included regions showing the least amount of sequence variation to maximize the sequence homology and thus silencing effect across all the members of the gene family (Supplemental Figure 1 of *AMS* ORF consensus sequence). The trichome specific promoter of the *cyp7av1* gene (Wang et al., 2011) was used to drive expression of the *AMS* gene silencing construct in-planta. *Agrobacterium tumefaciens* based transformation was used to generate three independent transgenic lines expressing the *cyp71av1::AMS*_RNAi construct in Artemis. Phenotypically the *AMS* silenced lines showed no significant differences when compared to NTCs in terms of height, branch number and leaf total dry weight (Supplemental Figure 3). Presence of the transgene was determined by PCR using primers designed to detect the *NPTII* selectable marker gene (Figure 3A). Q-RT-PCR revealed that there is a major reduction in steady state levels of *AMS* mRNA in all of the *AMS* silenced lines carrying the gene silencing construct (Figure 3B). This was mirrored by a dramatic decrease in artemisinin concentration in young, mature and dry leaves of the *AMS* silenced lines compared to the NTCs (Figure 3C). Amorpha 4-11-diene levels were found to be higher in young leaf tissue when compared to the mature and dry leaf material in both the NTC and AMS silenced lines with two of the lines showing a significant increase compared to the NTC control (Figure 4A). There was a significant reduction in all other intermediates downstream of amorpha 4-11-diene in the *AMS* silenced lines compared to NTC (Figure 4).

**Figure 5:**
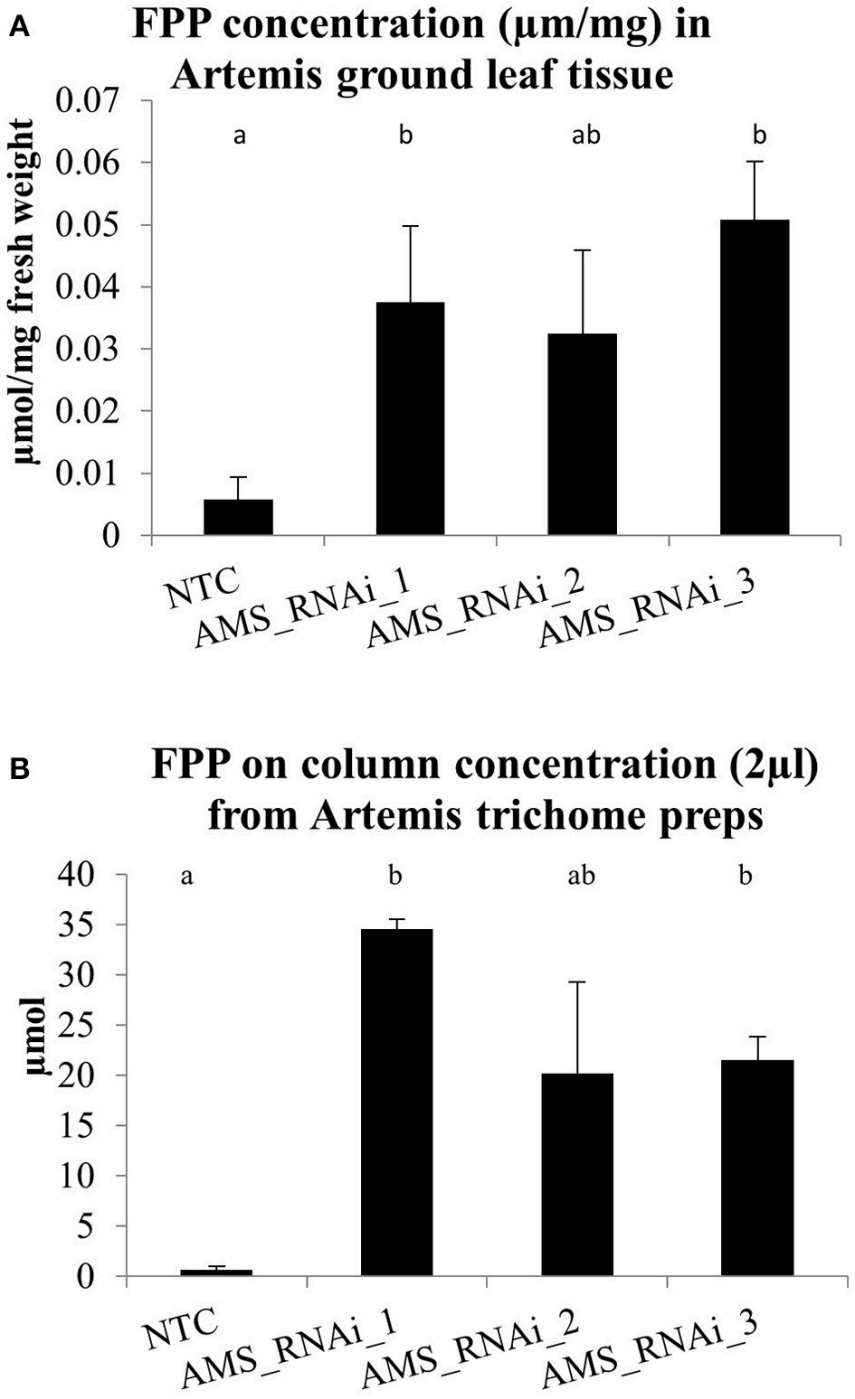
**(A)** FPP concentrations in μmol/g for ground young leaf material for the Artemis NTC and Artemis *AMS* silenced lines (concentrations were measured on Thermo Orbitrap Fusion Tribrid mass spectrometer). Error bars ±SD (NTC—*n* = 6, *AMS*_RNAi lines—*n* = 3). **(B)** On column FPP concentrations in μmol measured from isolated trichome extracts for Artemis NTC and Artemis *AMS* silenced lines (concentrations were measured on a Thermo LTQ Orbitrap Classic instrument, used in ion trap mode). Error bars ±SD (NTC—*n* = 6, *AMS*_RNAi lines—*n* = 3). Letters represent Bonferroni test results after ANOVA, groups not sharing letters indicate statistically significant differences (*p* ≤ 0.05).

Quantification of farnesyl diphosphate (FPP) in methanolic extracts from ground young leaf tips revealed a significant increase in *AMS* silenced lines compared to the NTCs (Figure 5A). This increase was also confirmed in isolated trichomes (Figure 5B).

### The consequence of FPP increases on known sesquiterpene synthase and squalene synthase gene expression

**Figure 6:**
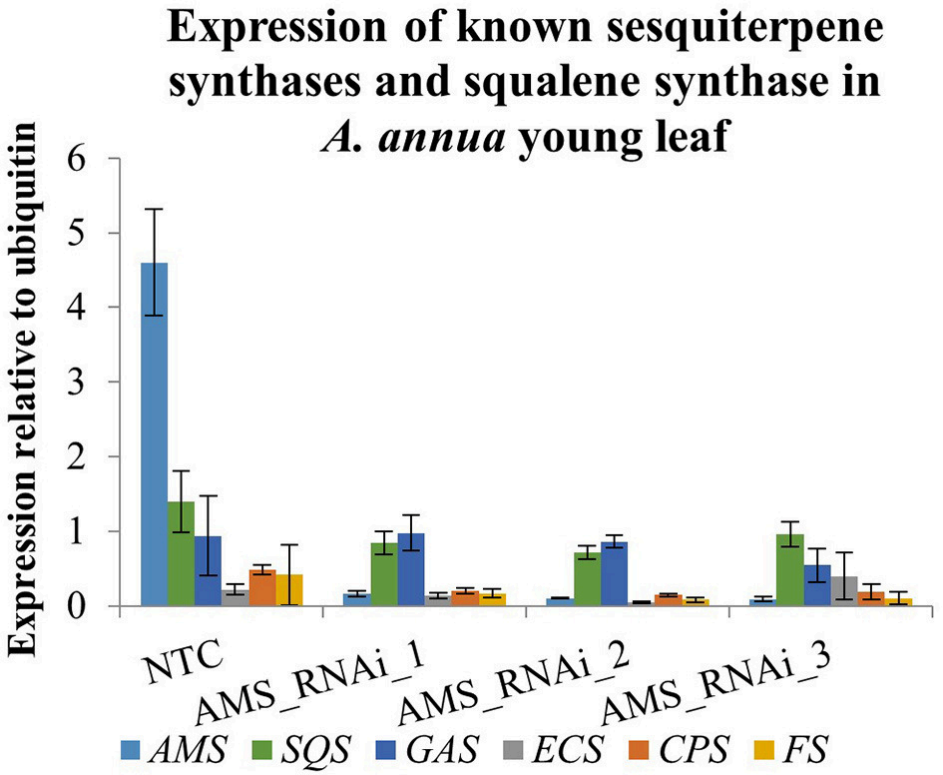
Expression of functionally characterized sesquiterpene synthase (*AMS, GAS, ECS, CPS*, and *FS*) and *SQUALENE SYNTHASE* (*SQS*) genes in young leaf tissue from NTC and *AMS* silenced lines. Error bars ±SD (NTC—*n* = 6, *AMS*_RNAi lines—*n* = 3).

The effect of silencing *AMS* on the expression of the other known sesquiterpene synthases and squalene synthase (detailed in Figure 1) was investigated by carrying out qRT-PCR on young leaf material (Figure 6). In the NTC the expression levels of *SQS, GAS, ECS, CPS*, and *FS* was found to be ~3- times lower than *AMS*. In the *AMS*_silenced lines *SQS* and *GAS* become the most highly expressed synthases although in comparison to the NTC they were not significantly increased. Comparison of expression of *SQS, GAS, ECS, CPS*, and *FS* between the NTC and *AMS*_silenced lines showed they are all lower in the latter except for *GAS* expression in the *AMS* silenced line, *AMS_* RNAi_1 and *ECS* expression in the *AMS* silenced *AMS*_RNAi_3. However, these slight differences in gene expression between NTC and the *AMS* silenced lines were not found to be significant.

### The downstream effect of increased FPP levels on sterol and sesquiterpene synthesis in artemis as quantified by GC-MS and UPLC

**Figure 7:**
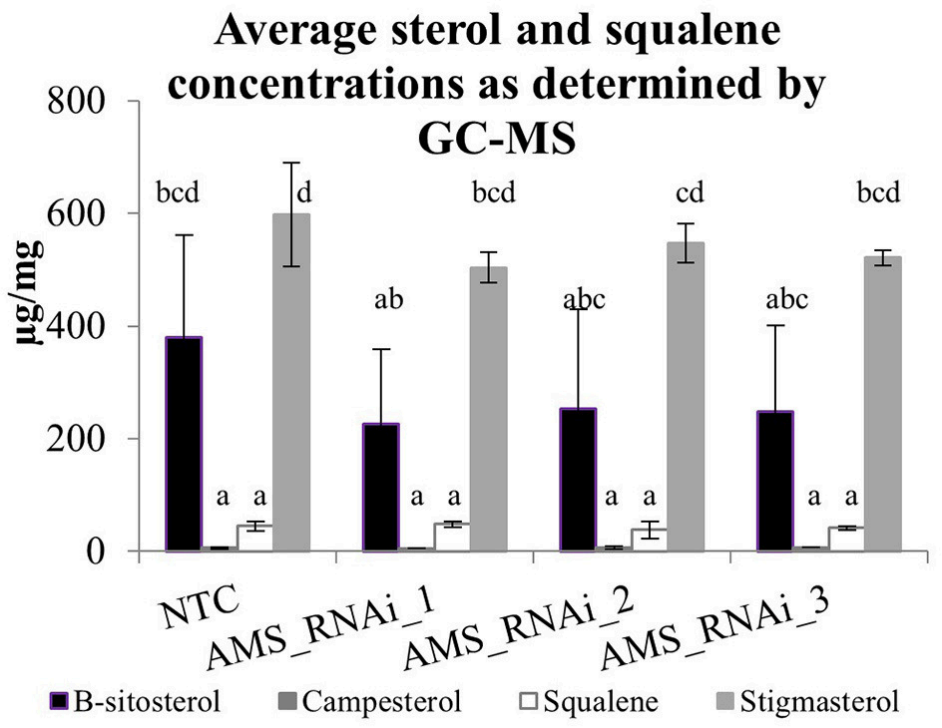
Average peak area from GC-MS for squalene and sterol compounds identified from dried pooled leaf material for NTC and *AMS* silenced lines. Error bars ±SD (NTC—*n* = 6, *AMS*_RNAi lines—*n* = 3). Letters represent Bonferroni test results after ANOVA, groups not sharing letters indicate statistically significant differences (*p* ≤ 0.05).

In *A. annua* FPP is a precursor for not only artemisinin and other sequiterpenes but also squalene and sterols and these could all therefore be additional sinks for FPP that does not flux into the artemisinin pathway via *AMS* (Figure 1). To determine if the silencing of *AMS* led to a redirection of FPP flux, squalene and sterol levels were quantified from dried leaf material by GC-MS. Squalene, stigmasterol, β-sitosterol and campesterol were identified in both the NTC and transformed lines (Figure 7). Stigmasterol and β-sitosterol were present at higher levels in comparison to squalene and campesterol but overall no significant differences were found between the NTC and the *AMS* silenced lines.

**Figure 8:**
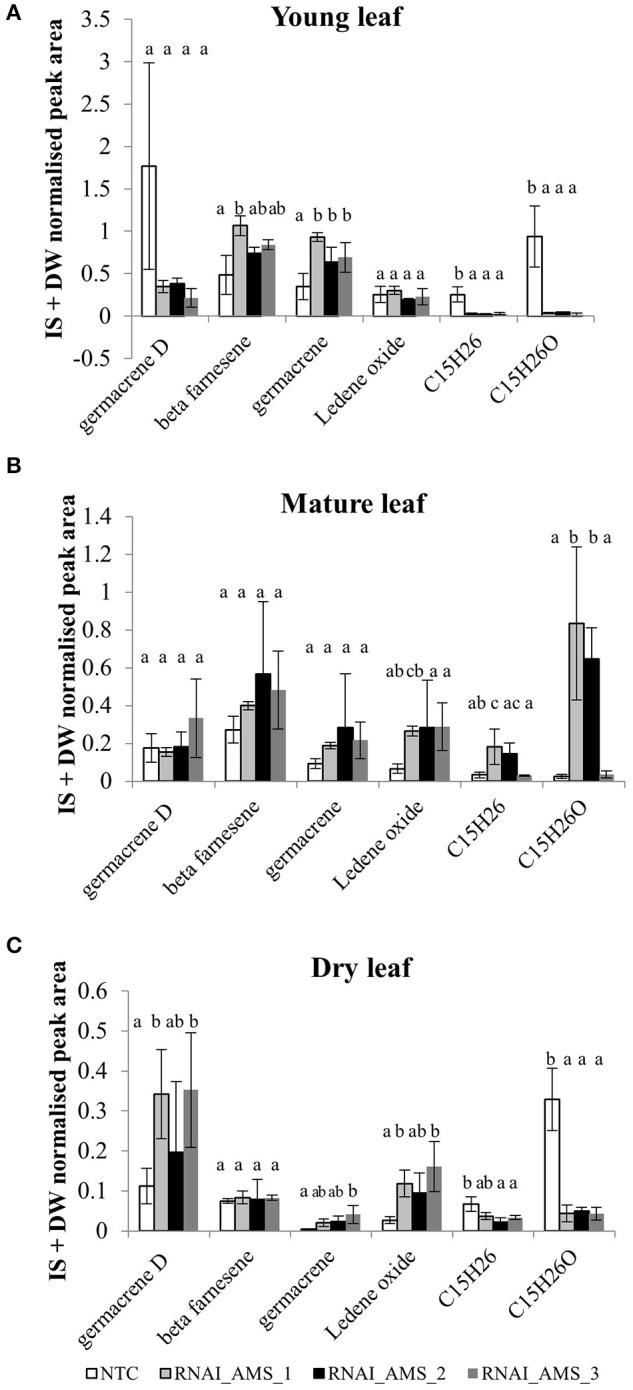
Internal standard (IS) and dry weight normalized peak area averages for sesquiterpene levels in **(A)** young leaf, **(B)** mature leaf, and **(C)** -pooled dried leaf material in NTC and *AMS* silenced lines. Error bars ±SD (NTC—*n* = 6, *AMS*_RNAi—*n* = 3). Letters represent Bonferroni test results after ANOVA, groups not sharing letters indicate statistically significant differences (*p* ≤ 0.05).

To determine if *AMS* silencing led to an increase in sesquiterpenes other than artemisinin, GC- and UPLC-MS analysis was carried out on fresh, young, and mature leaf material, and pooled dried leaf material. From the GC-MS analysis of NTC and transformed lines, 105 compounds were identified, 30 of which were sesquiterpenes. Comparisons between the leaf material sampled (young/mature/dried) revealed the level of sesquiterpenes to be higher in the young leaf samples in comparison to the mature and dried leaf material (Supplemental Table 1). Further statistical analysis to investigate differences between the 3 *AMS* silenced lines and the NTC found that for 17 of the sesquiterpene compounds levels were significantly higher in the NTC. Significant increases in the *AMS* silenced lines were found for only 6 sesquiterpene compounds (Supplemental Table 1). In young leaf material these were: beta-farnescene and germacrene; in mature leaf there were increases in 2 unknown compounds with putative C_15_H_24_ formulae, and in dried leaf material germacrene D and ledene oxide were significantly higher (Figure 8). For the other 7 sesquiterpene compounds no differences were observed.

**Figure 9:**
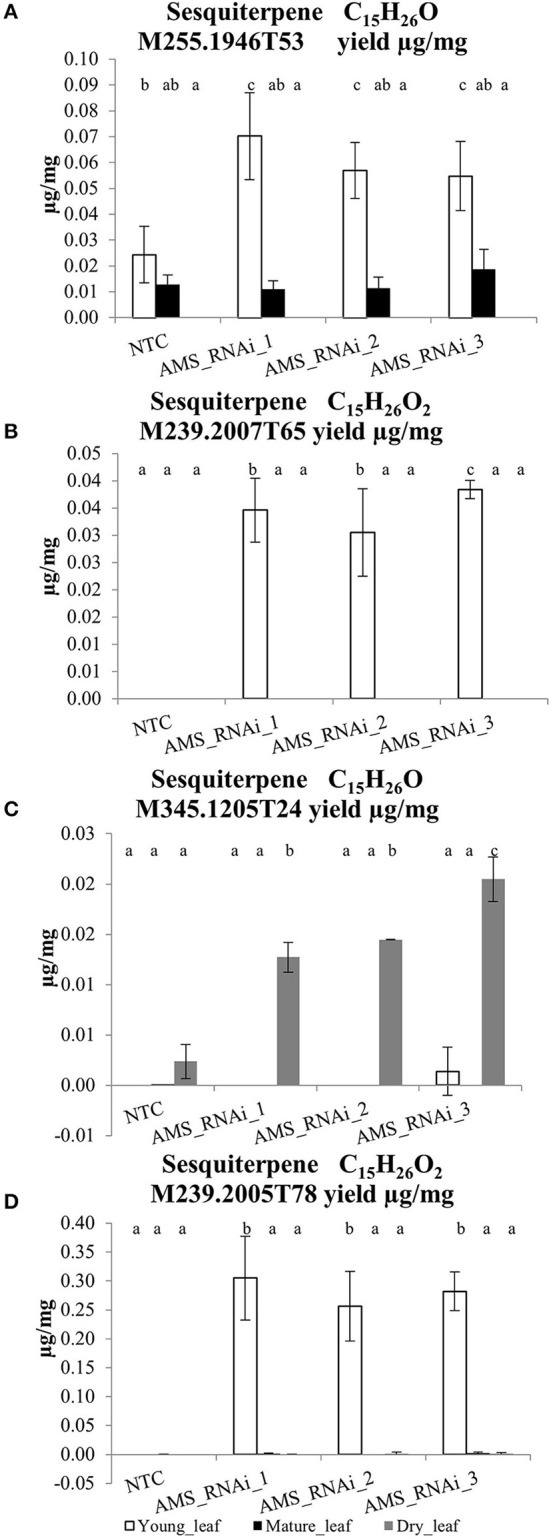
The concentration (μg/mg extracted dry weight) of putative novel sesquiterpene compounds in young and mature leaves and dried pooled leaf material for the *AMS* silenced lines and NTCs. **(A)** sesquiterpene M255.1946T53, **(B)** sesquiterpene M345.1205T24, **(C)** —M239.2007T65, and **(D)** —sesquiterpene M239.2005T78 Error bars ±SD (NTC—*n* = 6, *AMS*_RNAi lines—*n* = 3). Letters represent Bonferroni test results after ANOVA, groups not sharing letters indicate statistically significant differences (*p* ≤ 0.05).

As well as artemisinin and its associated compounds derived from the artemisinin pathway, the UPLC-MS analysis also identified four putative novel oxygenated sesquiterpene (C_15_H_24_O) compounds in the *AMS* silenced lines. These compounds were identified as being significantly increased although the levels at which they were present was very low, ranging from 0.03- to 0.4 μg/mg DW which is 100–10 times (respectively) lower than artemisinin levels (Figure 9). Putative sesquiterpenes: M255.1946T53 M239.2007T65 and M239.2005T78 were all found to be significantly increased in young leaf tissue in the transformed lines in comparison to the NTC. M345.1205T24 was found to be significantly increased in only the dried leaf tissue of the *AMS* silenced lines in comparison to the NTC.

## Discussion

### Silencing AMS leads to accumulation of the sesquiterpene precursor FPP in *A. annua* GSTs

In *A. anuua* the first committed step in the artemisinin pathway converting FPP to amorpha-4,11-diene is *AMS*. It was hypothesized that by blocking this step FPP would either accumulate or be channeled into the production of known or novel sesquiterpenes. Previous work had shown that *AMS* was not only highly expressed in the Artemis cultivar, but that recovered *AMS* gene sequences were polymorphic (Graham et al., 2010). This indicated that multiple copies of the gene could exist which we confirmed by qPCR (Figure 2). The high copy numbers for *AMS* present in the *A. annua cv*. Artemis could be linked to its high artemisinin yield, and its success as an elite hybrid for commercial production of artemisinin (Delabays et al., 2001). To effectively silence all the *AMS* copies two separate sections of the sequence were selected and joined to create an *AMS* specific sequence, this was driven by the *cyp71av1* trichome specific promoter (Wang et al., 2011). Stably transformed lines expressing the construct were achieved, with *AMS* expression reduced to less than 4% of the NTC. Artemisinin content was reduced by 95% alongside a reduction in all artemisinin-related compounds downstream of the *AMS*-catalyzed step.

One exception was amorpha-4,11-diene where levels were found to be significantly higher in young leaf tissue of the *AMS* silenced lines compared to the NTC (Figure 4A). No such increase was present in mature or dry leaves. The levels of amorpha-4,11-diene in *A. annua* are reported to be low as a consequence of artemisinin biosynthesis (Bouwmeester and Wallaart, 1999). Detection of this early step precursor in young leaves of NTC is consistent with previous findings (Czechowski et al., 2016) which suggest the pathway to artemisinin only becomes active as leaves mature. The increase in the *AMS* silenced lines is unexpected and the reason not obvious—but could relate to the metabolic sink being somehow further compromised as a result of the decreased *AMS*.

To establish the impact of silencing *AMS* on FPP levels we adapted a protocol from Nagel et al. (2014) that allowed us to quantify this important precursor for the first time in *A. annua*. We found that silencing of *AMS* led to a significant accumulation of FPP in young leaf tissue and this increase was also confirmed as being trichome specific by carrying out the same extraction on young leaf isolated GSTs (Figures 5A,B).

### The downstream effect of increased FPP levels on sterol and sesquiterpene synthesis in artemis as quantified by UPLC-MS and GC-MS

Increasing FPP by knocking down *AMS* had no effect on the expression of any of the other known sesquiterpene synthases genes or squalene synthase known to be expressed in either the GSTs or young leaf tissue (Figure 6). UPLC-MS and GC-MS analysis of leaf material was carried out to determine if the FPP accumulating in the AMS silenced lines was being redirected into sterol or sesquiterpene production. GC-MS analysis found no significant differences in squalene and sterol levels between the NTC and the *AMS* silenced lines (Figure 7) despite this pathway being considered the main competitor for FPP after artemisinin (Zhang et al., 2009). The GC-MS analysis also revealed very few changes in volatiles in the *AMS* silenced lines (Supplemental Table 1). Where significant differences were observed the magnitude, changes were very low (Figure 8). These results differ to the findings of Ma et al. (2015) who silenced *AMS* in a low artemisinin background (artemisinin yields of 0.025 μg/mg). Alongside reporting a decrease in artemisinin, they also found a significant increase in the levels of caryophyllene and copaene in their *AMS* silenced plants. The increase in these sesquiterpene compounds could be linked to the low artemisinin cultivar used for transformation having other active endogenous sesquiterpene synthases. In Artemis a high yielding cultivar the endogenous synthases would appear not to be as active in their ability to utilize the FPP made available away from artemisinin being silenced.

Although no differences were seen in known sesquiterpene levels in the AMS silenced lines, putative novel sesquiterpene compounds were detected and characterized by UPLC-MS. Significant differences were seen between Artemis NTCs and the *AMS* silenced lines for 4 putative sesquiterpene compounds although the concentrations were around 30 times lower than artemisinin. The compounds were also mainly identified in young leaf tissue suggesting that these compounds are not end products but rather are further converted as the leaf matures. Compound M345.1205T24 was an exception to the other 3 as it was found in dried leaf only. The very low concentrations of these novel compounds in available plant material ruled out structural determination attempts by NMR.

The lack of diversion of the accumulated FPP to other sesquiterpenes or sterols in the *AMS* silenced lines is somewhat surprising. One possible explanation for this is that the Artemis hybrid has been selected for high yield artemisinin and the flux of FPP may already be optimized to flow toward artemisinin production.

### Trichomes with elevated FPP as a potential production platform for high value sesquiterpenes

*A. annua* is already established as a very efficient crop plant for artemisinin production, with the potential to produce this high value chemical at a relatively low cost of less than $250 per kilogram. Disruption of *cyp71av1*, leading to novel arteannuin X accumulation demonstrated the plasticity of GST metabolism in *A. annua*, suggesting their potential as factories for new compound production (Czechowski et al., 2016). The GSTs provide an optimal environment for the synthesis of many natural products based on the availability of precursors, co-enzymes, mRNA and protein processing. In *A. annua* the problem of toxicity of some of these compounds is overcome as the GSTs can sequester them in the extracellular cavities of the trichome secretory cells. This coupled with the location of the GSTs on the surface of leaves is advantageous as the compounds are both contained and readily extractable.

By silencing *AMS* in a high artemisinin yielding *A. annua* cultivar we have significantly decreased the amount of artemisinin and related compounds produced in the GSTs. As a further result of the silencing we also show that the precursor FPP is accumulated in the GSTs and not catalyzed by endogenous synthases. The lack of production of novel compounds at significant amounts suggests that the elevated pool of GST localized FPP is either not available to or not utilized by other sesquiterpene, squalene synthase enzymes. Consequently, the *AMS* silenced lines may represent a platform for production of other high value compounds that require FPP as a precursor and for which genes encoding biosynthetic enzymes are known.

## Author contributions

TC, TMC, and IG designed the experiments; TC, TMC, CB, NS, JH, and DH performed the experiments; TMC, TC, CB, DH, and TL analyzed the data; TMC, TC, TL, and IAG wrote the manuscript; and all authors revised and approved the manuscript.

### Conflict of interest statement

The authors declare that the research was conducted in the absence of any commercial or financial relationships that could be construed as a potential conflict of interest.

## References

[B1] Bertea C. M. Voster A. Verstappen F. W. Maffei M. Beekwilder J. Bouwmeester H. J. (2006). Isoprenoid biosynthesis in *Artemisia annua*: cloning and heterologous expression of a germacrene a synthase from a glandular trichome cDNA library. Arch. Biochem. Biophys. 448, 3–12. 10.1016/j.abb.2006.02.026 16579958

[B2] Bleeker P. M. Mirabella R. Diergaarde P. J. VanDoorn A. Tissier A. Kant M. R.. (2012). Improved herbivore resistance in cultivated tomato with the sesquiterpene biosynthetic pathway from a wild relative. Proc. Natl. Acad. Sci. U.S.A. 109, 20124–20129. 10.1073/pnas.1208756109 23169639 PMC3523864

[B3] Bouwmeester H. J. Wallaart T. E. (1999). Amorpha-4, 11-diene synthase catalyses the first probable step in artemisinin biosynthesis. Phytochemistry 52, 843–854. 10.1016/S0031-9422(99)00206-X 10626375

[B4] Brown G. D. (2010). The biosynthesis of artemisinin (Qinghaosu) and the phytochemistry of *Artemisia annua* L. (Qinghao). Molecules 15, 7603–7698. 10.3390/molecules15117603 21030913 PMC6259225

[B5] Cai Y. Jia J.-W. Crock J. Lin Z.-X. Chen X.-Y. Croteau R. (2002). A cDNA clone for beta-caryophyllene synthase from *Artemisia annua*. Phytochemistry 61, 523–529. 10.1016/S0031-9422(02)00265-0 12409018

[B6] Chen J. L. Fang H. M. Ji Y. P. Pu G. Bin Guo Y. W.. (2011). Artemisinin biosynthesis enhancement in transgenic *Artemisia annua* plants by downregulation of the β-Caryophyllene synthase gene. Planta Med. 77, 1759–1765. 10.1055/s-0030-1271038 21509717

[B7] Croteau R. Kutchan T. M. Lewis N. G. (2000). Secondary metabolites. Biochem. Mol. Biol. Plants 7, 1250–1318. 10.1016/j.phytochem.2011.10.011

[B8] Czechowski T. Larson T. R. Catania T. M. Harvey D. Brown G. D. Graham I. A. (2016). *Artemisia annua* mutant impaired in artemisinin synthesis demonstrates importance of nonenzymatic conversion in terpenoid metabolism. *Proc. Natl. Acad. Sci*. U.S.A. 113, 15150–15155. 10.1073/pnas.1611567113 PMC5206525 27930305

[B9] Delabays N. Simonnet X. Gaudin M. (2001). The genetics of artemisinin content in *Artemisia annua* L. and the breeding of high yielding cultivars. *Curr. Med*. Chem. 8, 1795–1801. 10.2174/0929867013371635 11772351

[B10] Duke M. V. Paul R. N. Elsohly H. N. Sturtz G. Duke S. O. (1994). Localization of artemisinin and artemisitene in foliar tissues of glanded and glandless biotypes of *Artemisia annua* L. Int. J. Plant Sci. 155, 365–372. 10.1086/297173

[B11] Duke S. O. Paul R. N. (1993). Development and fine structure of the glandular trichomes of *Artemisia annua* L. Int. J. Plant Sci. 154, 107–118. 10.1086/297096

[B12] Ferreira J. F. S. Janick J. (1995). Floral morphology of *Artemisia annua* with special reference to trichomes. Int. J. Plant Sci. 156, 807–815. 10.1086/297304

[B13] Ferreira J. F. S. Laughlin J. C. Delabays N. de Magalhães P. M. (2005). Cultivation and genetics of *Artemisia annua* L. for increased production of the antimalarial artemisinin. Plant Genet. Resour. Charact. Util. 3, 206–229. 10.1079/PGR200585

[B14] Graham I. A. Besser K. Blumer S. Branigan C. A. Czechowski T. Elias. (2010). The genetic map of *Artemisia annua* L. identifies loci affecting yield of the antimalarial Drug Artemisinin. Science. 327, 328–331. 10.1126/science.1182612 20075252

[B15] Han J. L. Liu B. Y. Ye H. C. Wang H. Li Z. Q. Li G. F. (2006). Effects of overexpression of the endogenous farnesyl diphosphate synthase on the artemisinin content in *Artemisia annua* L. J. Integr. Plant Biol. 48, 482–487. 10.1111/j.1744-7909.2006.00208.x

[B16] Han J. Wang H. Kanagarajan S. Hao M. Lundgren A. Brodelius P. E. (2016). Promoting artemisinin biosynthesis in *Artemisia annua* plants by substrate channeling. Mol. Plant. 9, 946–948. 10.1016/j.molp.2016.03.004 26995295

[B17] Huchelmann A. Boutry M. Hachez C. (2017). Plant glandular trichomes: natural cell factories of high biotechnological interest. Plant Physiol. 175, 6–22. 10.1104/pp.17.00727 28724619 PMC5580781

[B18] Ikram N. K. B. K. Simonsen H. T. (2017). A review of biotechnological artemisinin production in plants. Front. Plant Sci. 8:1966. 10.3389/fpls.2017.01966 29187859 PMC5694819

[B19] Kortbeek R. W. Xu J. Ramirez A. Spyropoulou E. Diergaarde P. Otten-Bruggeman M.. (2016). Engineering of tomato glandular trichomes for the production of specialized metabolites. Methods Enzymol. 576, 305–331. 10.1016/bs.mie.2016.02.014 27480691

[B20] Lv Z. Zhang F. Pan Q. Fu X. Jiang W. Shen Q.. (2016). Branch pathway blocking in *Artemisia annua* is a useful method for obtaining high yield artemisinin. Plant Cell Physiol. 57, 588–602. 10.1093/pcp/pcw014 26858285

[B21] Ma C. Wang H. Lu X. Wang H. Xu G. Liu B. (2009). Terpenoid metabolic profiling analysis of transgenic *Artemisia annua* L. by comprehensive two-dimensional gas chromatography time-of-flight mass spectrometry. Metabolomics 5, 497–506. 10.1007/s11306-009-0170-6

[B22] Ma D. M. Wang Z. Wang L. Alejos-Gonzales F. Sun M. A. Xie D. Y. (2015). A Genome-wide scenario of terpene pathways in self-pollinated *Artemisia annua*. Mol. Plant 8, 1580–1598. 10.1016/j.molp.2015.07.004 26192869

[B23] Mercke P. Crock J. Croteau R. Brodelius P. E. (1999). Cloning, expression, and characterization of epi-cedrol synthase, a sesquiterpene cyclase from *Artemisia annua* L. Arch. Biochem. Biophys. 369, 213–222. 10.1006/abbi.1999.1358 10486140

[B24] Nagel R. Berasategui A. Paetz C. Gershenzon J. Schmidt A. (2014). Overexpression of an isoprenyl diphosphate synthase in spruce leads to unexpected terpene diversion products that function in plant defense. Plant Physiol. 164, 555–569. 10.1104/pp.113.228940 24346420 PMC3912089

[B25] Olofsson L. Engström A. Lundgren A. Brodelius P. E. (2011). Relative expression of genes of terpene metabolism in different tissues of *Artemisia annua* L. BMC Plant Biol. 11:45. 10.1186/1471-2229-11-45 21388533 PMC3063820

[B26] Olofsson L. Lundgren A. Brodelius P. E. (2012). Trichome isolation with and without fixation using laser microdissection and pressure catapulting followed by RNA amplification: expression of genes of terpene metabolism in apical and sub-apical trichome cells of *Artemisia annua* L. Plant Sci. 183, 9–13. 10.1016/j.plantsci.2011.10.019 22195571

[B27] Olsson M. E. Olofsson L. M. Lindahl A. L. Lundgren A. Brodelius M. Brodelius P. E. (2009). Localization of enzymes of artemisinin biosynthesis to the apical cells of glandular secretory trichomes of *Artemisia annua* L. Phytochemistry 70, 1123–1128. 10.1016/j.phytochem.2009.07.009 19664791

[B28] Paddon C. J. Keasling J. D. (2014). Semi-synthetic artemisinin: a model for the use of synthetic biology in pharmaceutical development. Nat. Rev. Microbiol. 12, 355–367. 10.1038/nrmicro3240 24686413

[B29] Peplow M. (2016). Synthetic biology's first malaria drug meets market resistance. Nature 530, 389–390. 10.1038/530390a 26911755

[B30] Picaud S. Brodelius M. Brodelius P. E. (2005). Expression, purification and characterization of recombinant (E)-beta-farnesene synthase from *Artemisia annua*. Phytochemistry 66, 961–967. 10.1016/j.phytochem.2005.03.027 15896363

[B31] Pulice G. Pelaz S. Matías-Hernández L. (2016). Molecular farming in *Artemisia annua*, a promising approach to improve anti-malarial drug production. Front. Plant Sci. 7:329. 10.3389/fpls.2016.00329 27047510 PMC4796020

[B32] Soetaert S. S. Van Neste C. M. Vandewoestyne M. L. Head S. R. Goossens A. Van Nieuwerburgh F. C.. (2013). Differential transcriptome analysis of glandular and filamentous trichomes in *Artemisia annua*. BMC Plant Biol. 13:220. 10.1186/1471-2229-13-220 24359620 PMC3878173

[B33] Tang K. Shen Q. Yan T. Fu X. (2014). Transgenic approach to increase artemisinin content in *Artemisia annua* L. Plant Cell Rep. 33, 605–615. 10.1007/s00299-014-1566-y 24413765

[B34] Tang K. X. Wang Y. Y. Tang Y. L. Chen D. (2011). Method of utilizing the pts gene and RNA interference of the ads gene to increase patchouli alcohol content in *Artemisia annua* l. U.S. Patent No 0300546A1.

[B35] Tissier A. Sallaud C. Rontein D. (2012). Tobacco trichomes as a platform for terpenoid biosynthesis engineering, in Isoprenoid Synthesis in Plants and Microorganisms, eds Bach T. Rohmer M. (New York, NY: Springer), 271–283.

[B36] Van Noorden R. (2010). Demand for malaria drug soars. Nature 466, 672–673 10.1038/466672a 20686539

[B37] Wang W. Wang Y. Zhang Q. Qi Y. Guo D. (2009). Global characterization of *Artemisia annua* glandular trichome transcriptome using 454 pyrosequencing. BMC Genomics 10:465. 10.1186/1471-2164-10-465 19818120 PMC2763888

[B38] Wang Y. Yang K. Jing F. Li M. Deng T. Huang R.. (2011). Cloning and characterization of trichome-specific promoter of cpr71av1 gene involved in artemisinin biosynthesis in *Artemisia annua* L. Mol. Biol. 45, 751–758. 10.1134/S0026893311040145 22393777

[B39] Watson J. M. Fusaro A. F. Wang M. Waterhouse P. M. (2005). RNA silencing platforms in plants. FEBS Lett. 579, 5821–6009. 10.1016/j.febslet.2005.08.014 16139270

[B40] Weathers P. J. Elkholy S. Wobbe K. K. (2006). Artemisinin: the biosynthetic pathway and its regulation in *Artemisia annua*, a terpenoid-rich species. Vitr. Cell. Dev. Biol. Plant 42, 309–317. 10.1079/IVP2006782

[B41] Wu S. Schalk M. Clark A. Miles R. B. Coates R. Chappell J. (2006). Redirection of cytosolic or plastidic isoprenoid precursors elevates terpene production in plants. Nat. Biotechnol. 24, 1441–1447. 10.1038/nbt1251 17057703

[B42] Xie D. Y. Ma D. M. Judd R. Jones A. L. (2016). Artemisinin biosynthesis in *Artemisia annua* and metabolic engineering: questions, challenges, and perspectives. Phytochem. Rev. 15, 1093–1114. 10.1007/s11101-016-9480-2

[B43] Yang R. Y. Feng L. L. Yang X. Q. Yin L. L. Xu X. L. Zeng Q. P. (2008). Quantitative transcript profiling reveals down-regulation of a sterol pathway relevant gene and overexpression of artemisinin biogenetic genes in transgenic *Artemisia annua* plants. Planta Med. 74, 1510–1516. 10.1055/s-2008-1081333 18816428

[B44] Yu G. Pichersky E. (2014). Heterologous expression of methylketone synthase1 and methylketone synthase2 leads to production of methylketones and myristic acid in transgenic plants. Plant Physiol. 164, 612–622. 10.1104/pp.113.228502 24390393 PMC3912093

[B45] Zhang L. Jing F. Li F. Li M. Wang Y. Wang G.. (2009). Development of transgenic *Artemisia annua* (Chinese wormwood) plants with an enhanced content of artemisinin, an effective anti-malarial drug, by hairpin-RNA-mediated gene silencing. Biotechnol. Appl. Biochem. 52, 199–207. 10.1042/BA20080068 18564056

[B46] Zhang Y. Nowak G. Reed D. W. Covello P. S. (2011). The production of artemisinin precursors in tobacco. Plant Biotechnol. J. 9, 445–454. 10.1111/j.1467-7652.2010.00556.x 20723135

