# Supplementary material for: Silencing amorpha-4,11-diene synthase Genes in Artemisia annua Leads to FPP Accumulation
Source: Front Plant Sci. 2018 May 29;9:547. doi: 10.3389/fpls.2018.00547 (PMC5986941; doi:10.3389/fpls.2018.00547)
Supplement: Supplementary file 1 [file Table_1.DOCX]

|  | **Dried leaf** | | | | **Sig.** | **Mature_leaf** | | | | **Sig.** | **Young_leaf** | | | | **Sig.** |
| --- | --- | --- | --- | --- | --- | --- | --- | --- | --- | --- | --- | --- | --- | --- | --- |
|  | **NTC** | ***AMS*_** | ***AMS*_** | ***AMS*_** |  | **NTC** | ***AMS*_** | ***AMS*_** | ***AMS*_** |  | **NTC** | ***AMS_*** | ***AMS*_** | ***AMS*_** |  |
|  |  | **RNAi_1** | **RNAi_2** | **RNAi_3** |  |  | **RNAi_1** | **RNAi_2** | **RNAi_3** |  |  | **RNAi_1** | **RNAi_2** | **RNAi_3** |  |
| M91T623_C_15_H_24_ germacrene D pk1 | **0.073** | **0.019** | **0.192** | **0.067** | **NS** | **2.132** | **1.524** | **2.846** | **4.035** | **NS** | **2.48** | **1.226** | **1.06** | **1.041** | **NS** |
|  | *0.067* | *0.02* | *0.284* | *0.088* |  | *0.886* | *0.109* | *0.812* | *3.275* |  | *1.7* | *0.265* | *0.252* | *0.467* |  |
| M43T679_C_15_H_24_ germacrene D pk3 | **0.112 (a)** | **0.342 (b)** | **0.196(ab)** | **0.353(b)** | ***** | **0.176** | **0.154** | **0.183** | **0.333** | **NS** | **1.767** | **0.346** | **0.381** | **0.214** | **NS** |
|  | *0.045* | *0.111* | *0.178* | *0.144* |  | *0.075* | *0.024* | *0.078* | *0.208* |  | *1.219* | *0.072* | *0.067* | *0.111* |  |
| M39T689_C_15_H_24_ germacrene A | **0.09** | **0.119** | **0.102** | **0.123** | **NS** | **0.539** | **0.275** | **0.393** | **0.28** | **NS** | **2.076** | **0.661** | **0.606** | **0.573** | **NS** |
|  | *0.018* | *0.004* | *0.02* | *0.011* |  | *0.113* | *0.041* | *0.252* | *0.164* |  | *1.248* | *0.011* | *0.026* | *0.062* |  |
| M41T693_1_C_15_H_24_ guaiene | **0.121** | **0.157** | **0.128** | **0.152** | **NS** | **0.361** | **0.465** | **0.413** | **0.361** | **NS** | **1.594** | **0.882** | **0.844** | **0.784** | **NS** |
|  | *0.024* | *0.015* | *0.03* | *0.014* |  | *0.067* | *0.038* | *0.239* | *0.167* |  | *0.583* | *0.087* | *0.059* | *0.109* |  |
| M75T692_C_15_H_24_ unknown | **0.034** | **0.039** | **0.025** | **0.019** | **NS** | **0.767** | **0.456** | **0.672** | **0.552** | **NS** | **2.085(b)** | **0.742(a)** | **0.891(a)** | **0.772(a)** | ****** |
|  | *0.017* | *0.007* | *0.013* | *0.002* |  | *0.128* | *0.026* | *0.374* | *0.218* |  | *0.665* | *0.026* | *0.25* | *0.088* |  |
| M41T603_C_15_H_24_ beta farnesene | **0.075** | **0.084** | **0.079** | **0.083** | **NS** | **0.272** | **0.399** | **0.567** | **0.482** | **NS** | **0.484(a)** | **1.067(b)** | **0.741(ab)** | **0.840(ab)** | ****** |
|  | *0.006* | *0.016* | *0.05* | *0.007* |  | *0.071* | *0.021* | *0.383* | *0.206* |  | *0.228* | *0.115* | *0.068* | *0.058* |  |
| M91T599_C_15_H_24_ trans-caryophyllene pk1 | **0.039** | **0.039** | **0.089** | **0.071** | **NS** | **0.465** | **0.589** | **0.941** | **0.709** | **NS** | **0.323** | **0.411** | **0.276** | **0.354** | **NS** |
|  | *0.02* | *0.028* | *0.088* | *0.041* |  | *0.133* | *0.057* | *0.604* | *0.251* |  | *0.133* | *0.038* | *0.062* | *0.059* |  |
| M161T775_C_15_H_24_ Germacrene D pk4 | **0.175(b)** | **0.016(a)** | **0.010(a)** | **0.010(a)** | ******* | **0.518(b)** | **0.026(a)** | **0.011(a)** | **0.012(a)** | ******* | **2.235(a)** | **0.061(ab)** | **0.015(a)** | **0.016(ab)** | ***** |
|  | *0.049* | *0.005* | *0.004* | *0.002* |  | *0.1* | *0.006* | *0.007* | *0.005* |  | *1.189* | *0.018* | *0.002* | *0.003* |  |
| M79T626_C_15_H_24_ germacrene | **0.004(a)** | **0.020(ab)** | **0.023(ab)** | **0.041(b)** | ****** | **0.091** | **0.189** | **0.284** | **0.217** | **NS** | **0.35(a)** | **0.929(b)** | **0.640(b)** | **0.693(b)** | ****** |
|  | *0.001* | *0.01* | *0.014* | *0.022* |  | *0.026* | *0.017* | *0.282* | *0.097* |  | *0.154* | *0.051* | *0.173* | *0.176* |  |
| ^1^M91T697_C_15_H_24_O | **0.041** | **0.031** | **0.046** | **0.044** | **NS** | **0.13** | **0.075** | **0.125** | **0.086** | **NS** | **0.34** | **0.327** | **0.257** | **0.274** | **NS** |
|  | *0.015* | *0.028* | *0.041* | *0.034* |  | *0.048* | *0.013* | *0.102* | *0.032* |  | *0.183* | *0.045* | *0.019* | *0.085* |  |
| M43T778_C_15_H_24_O unknown | **0.408** | **0.319** | **0.202** | **0.31** | **NS** | **0.275(b)** | **0.059(a)** | **0.073(a)** | **0.057(a)** | ******* | **2.246(b)** | **0.090(a)** | **0.095(a)** | **0.085(a)** | ****** |
|  | *0.247* | *0.041* | *0.075* | *0.027* |  | *0.064* | *0.01* | *0.051* | *0.022* |  | *1.055* | *0.015* | *0.035* | *0.007* |  |
| M187T681_C_15_H_24_O Ledene oxide | **0.027(a)** | **0.118(b)** | **0.095(ab)** | **0.161(b)** | ****** | **0.066** | **0.264** | **0.283** | **0.288** | **NS** | **0.254** | **0.303** | **0.193** | **0.228** | **NS** |
|  | *0.008* | *0.033* | *0.05* | *0.062* |  | *0.026* | *0.027* | *0.251* | *0.126* |  | *0.098* | *0.046* | *0.017* | *0.094* |  |
| M41T801_C_15_H_24_O unknown | **0.0878(b)** | **0.022(a)** | **0.010(a)** | **0.011(a)** | ******* | **0.251(a)** | **0.031(b)** | **0.027(b)** | **0.013(b)** | ******* | **0.835(b)** | **0.069(a)** | **0.048(a)** | **0.047(a)** | ****** |
|  | *0.024* | *0.005* | *0.006* | *0.003* |  | *0.06* | *0.01* | *0.028* | *0.007* |  | *0.335* | *0.002* | *0.008* | *0.001* |  |
| M93T645_C_15_H_24_ alpha humulene pk2 | **0.007** | **0.008** | **0.009** | **0.009** | **NS** | **0.012** | **0.008** | **0.01** | **0.006** | **NS** | **0.398(a)** | **0.032(ab)** | **0.018(a)** | **0.016(a)** | ***** |
|  | *0.003* | *0.002* | *0.005* | *0.002* |  | *0.004* | *0.001* | *0.006* | *0.002* |  | *0.227* | *0.002* | *0.001* | *0.001* |  |
| ^2^M187T745_C_15_H_24_ O | **0.004(b)** | **0.002(a)** | **0.002(a)** | **0.001(a)** | ****** | **0.007(b)** | **0.001(a)** | **0.001(a)** | **0.002(a)** | ******* | **0.702(b)** | **0.014(a)** | **0.009(a)** | **0.005(a)** | ****** |
|  | *0.001* | *0* | *0* | *0* |  | *0.002* | *0.001* | *0* | *0* |  | *0.333* | *0.003* | *0.002* | *0.001* |  |
| M29T685_C_15_H_24_ valencene | **0.061** | **0.111** | **0.065** | **0.049** | **NS** | **0.034** | **0.027** | **0.034** | **0.031** | **NS** | **0.426(b)** | **0.017(a)** | **0.014(a)** | **0.011(a)** | ****** |
|  | *0.019* | *0.064* | *0.044* | *0.02* |  | *0.009* | *0.007* | *0.029* | *0.019* |  | *0.202* | *0.004* | *0.001* | *0.003* |  |
| M59T675_C_15_H_24_ germacrene D pk2 | **0.002** | **0.002** | **0.002** | **0.002** | **NS** | **0.008(b)** | **0.002(a)** | **0.003(a)** | **0.004(a)** | ***** | **0.599(b)** | **0.021(a)** | **0.008(a)** | **0.008(a)** | ****** |
|  | *0.001* | *0.001* | *0.002* | *0.001* |  | *0.003* | *0.001* | *0.002* | *0.001* |  | *0.28* | *0.005* | *0.001* | *0.002* |  |
| M91T722_C_15_H_24_O unknown | **0.008** | **0.008** | **0.008** | **0.006** | **NS** | **0.081** | **0.027** | **0.045** | **0.023** | **NS** | **0.291(a)** | **0.090(ab)** | **0.103(ab)** | **0.080(a)** | ***** |
|  | *0.002* | *0.003* | *0.003* | *0.004* |  | *0.027* | *0.002* | *0.052* | *0.009* |  | *0.119* | *0.019* | *0.002* | *0.002* |  |
| M39T566_C_15_H_24_ copaene pk1 | **0.011** | **0.012** | **0.011** | **0.019** | **NS** | **0.144(b)** | **0.035(a)** | **0.041(a)** | **0.039(a)** | ******* | **0.423(b)** | **0.147(a)** | **0.134(a)** | **0.114(a)** | ****** |
|  | *0.004* | *0.004* | *0.003* | *0.012* |  | *0.041* | *0.001* | *0.027* | *0.019* |  | *0.147* | *0.012* | *0.005* | *0.015* |  |
| M43T749_C_15_H_24_ unknown | **0.038** | **0.024** | **0.029** | **0.028** | **NS** | **0.048** | **0.04** | **0.041** | **0.04** | **NS** | **0.385** | **0.133** | **0.167** | **0.136** | **NS** |
|  | *0.008* | *0.005* | *0.01* | *0.004* |  | *0.014* | *0.007* | *0.042* | *0.022* |  | *0.201* | *0.019* | *0.006* | *0.003* |  |
| M105T579_C_15_H_24_ copaene pk2 | **0.025** | **0.018** | **0.024** | **0.029** | **NS** | **0.073** | **0.078** | **0.143** | **0.113** | **NS** | **0.063** | **0.065** | **0.05** | **0.065** | **NS** |
|  | *0.007* | *0.007* | *0.018* | *0.01* |  | *0.018* | *0.004* | *0.1* | *0.035* |  | *0.024* | *0.006* | *0.016* | *0.016* |  |
| M138T763_C_15_H_24_O unknown | **0.233(b)** | **0.152(a)** | **0.144(a)** | **0.142(a)** | ******* | **0.087** | **0.035** | **0.058** | **0.049** | **NS** | **0.168** | **0.092** | **0.152** | **0.144** | **NS** |
|  | *0.023* | *0.016* | *0.024* | *0.046* |  | *0.045* | *0.005* | *0.027* | *0.021* |  | *0.032* | *0.016* | *0* | *0.076* |  |
| M161T615_C_15_H_24_ alpha humulene pk1 | **0.002** | **0.002** | **0.002** | **0.001** | **NS** | **0.1** | **0.071** | **0.104** | **0.104** | **NS** | **0.163** | **0.119** | **0.168** | **0.143** | **NS** |
|  | *0* | *0.001* | *0.001* | *0.001* |  | *0.022* | *0.009* | *0.042* | *0.042* |  | *0.039* | *0.011* | *0.059* | *0.02* |  |
| M43T836_C_15_H_24_O unknown | **0.116(c )** | **0.064(ab)** | **0.021(a)** | **0.083(bc)** | ****** | **0.044(b)** | **0.016(a)** | **0.013(a)** | **0.018(a)** | ******* | **0.038** | **0.007** | **0.023** | **0.01** | **NS** |
|  | *0.028* | *0.037* | *0.002* | *0.005* |  | *0.008* | *0.005* | *0.004* | *0.011* |  | *0.02* | *0.005* | *0.003* | *0.003* |  |
| M119T674_C_15_H_24_ gamma curcumene | **0.002** | **0.003** | **0.003** | **0.003** | **NS** | **0.002** | **0.001** | **0.001** | **0.001** | **NS** | **0.361(a)** | **0.004(ab)** | **0.001(ab)** | **0.001(a)** | ***** |
|  | *0.001* | *0.001* | *0.002* | *0.001* |  | *0.001* | *0.001* | *0.001* | *0.001* |  | *0.195* | *0* | *0.001* | *0* |  |
| M191T625_C_15_H_24_  trans-caryophyllene pk2 | **0.0005(b)** | **0.0002(a)** | **0.0002(a)** | **0.0003(a)** | ***** | **0.03** | **0.024** | **0.032** | **0.029** | **NS** | **0.042** | **0.029** | **0.039** | **0.034** | **NS** |
|  | *0* | *0* | *0* | *0* |  | *0.007* | *0.002* | *0.014* | *0.011* |  | *0.009* | *0.002* | *0.009* | *0.004* |  |
| M91T660_C_15_H_22_ spathulenol | **0.097** | **0.121** | **0.108** | **0.133** | **NS** | **0.077** | **0.042** | **0.046** | **0.06** | **NS** | **0.087** | **0.08** | **0.073** | **0.077** | **NS** |
|  | *0.009* | *0.035* | *0.038* | *0.032* |  | *0.028* | *0.009* | *0.026* | *0.005* |  | *0.027* | *0.012* | *0.01* | *0.002* |  |
| M43T781_C_15_H_26_O_2_ unknown | **0.093** | **0.156** | **0.09** | **0.152** | **NS** | **0.096** | **0.15** | **0.145** | **0.078** | **NS** | **0.435(b)** | **0.127(a)** | **0.158(a)** | **0.167(a)** | ******* |
|  | *0.056* | *0.015* | *0.043* | *0.009* |  | *0.049* | *0.034* | *0.019* | *0.019* |  | *0.096* | *0.025* | *0.015* | *0.021* |  |
| M41T793_C_15_H_26_ unknown | **0.067(b)** | **0.037(ab)** | **0.022(a)** | **0.033(a)** | ****** | **0.033(ab)** | **0.183(c)** | **0.145(ac)** | **0.030(a)** | ****** | **0.255(b)** | **0.027(a)** | **0.021(a)** | **0.029(a)** | ****** |
|  | *0.019* | *0.008* | *0.011* | *0.005* |  | *0.016* | *0.094* | *0.055* | *0.004* |  | *0.092* | *0.01* | *0.006* | *0.014* |  |
| M41T820_C_15_H_26_O unknown | **0.329(b)** | **0.044(a)** | **0.049(a)** | **0.043(a)** | ******* | **0.026(a)** | **0.834(b)** | **0.647(b)** | **0.035(a)** | ******* | **0.939(b)** | **0.035(a)** | **0.046(a)** | **0.017(a)** | ****** |
|  | *0.078* | *0.021* | *0.009* | *0.016* |  | *0.011* | *0.404* | *0.164* | *0.017* |  | *0.359* | *0.009* | *0.006* | *0.02* |  |

Supplemental table 1 – Average values for the GC-MS identified sesquiterpene compounds in the Artemis transformed lines and NTC. Bold numbers represent averages calculated for the transformed lines (n=3) and NTC (n=6) numbers in italics = standard deviations. The sig. column is the p-values from ANOVA to compare the difference between transformed and NTC data. NS = non-significant, *=<0.05 **=<0.01 ***=<0.001 lower case letters indicate significant increases identified in transformed lines in comparison to the NTC as determined by Bonferroni (p=<0.05)

^1^M91T697_C_15_H_24_O (1R,7S,E)-7-Isopropyl-4,10-dimethylenecyclodec-5-enol

^2^M187T745_C_15_H_24_ ODimethyl-3-(prop-1-en-2-yl)-1,2,3,4,4a,5,6,7-octahydronaphthalen-1-ol
