# Supplementary figures and images for: Silencing amorpha-4,11-diene synthase Genes in Artemisia annua Leads to FPP Accumulation
Source: Front Plant Sci. 2018 May 29;9:547. doi: 10.3389/fpls.2018.00547 (PMC5986941; doi:10.3389/fpls.2018.00547)

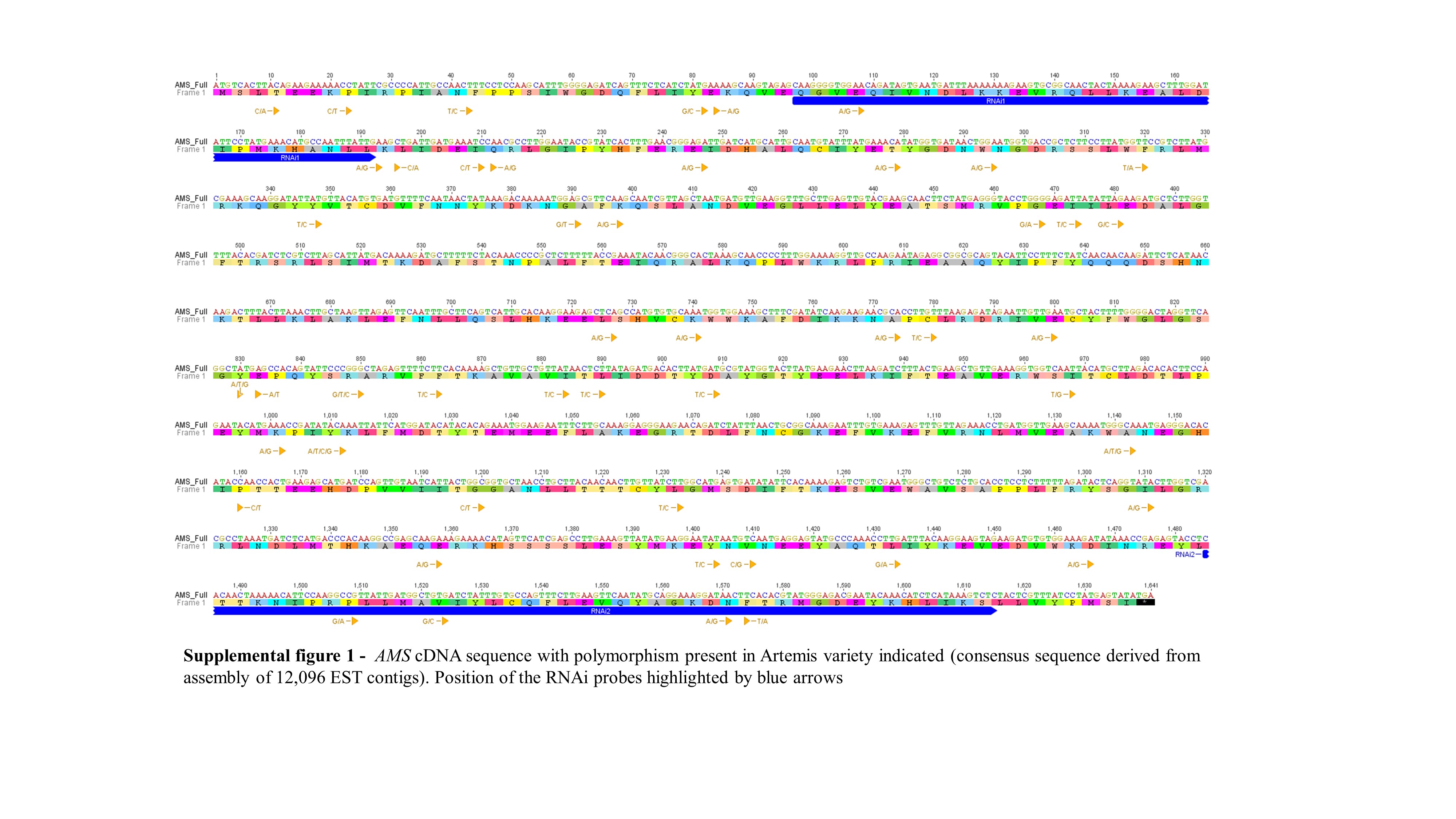

Supplement: Supplementary file 2 [file Image_1.jpeg]

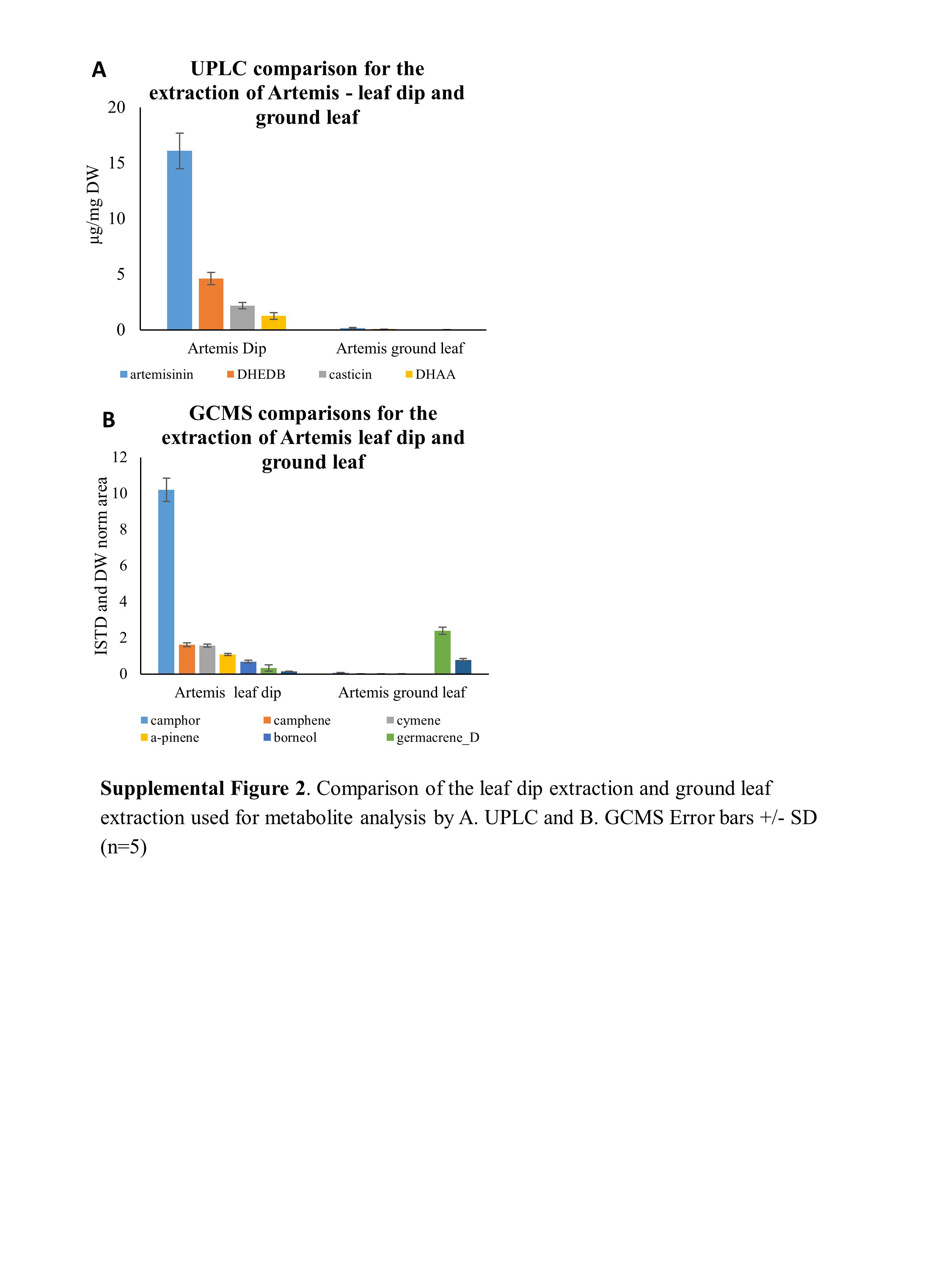

Supplement: Supplementary file 3 [file Image_2.jpg]

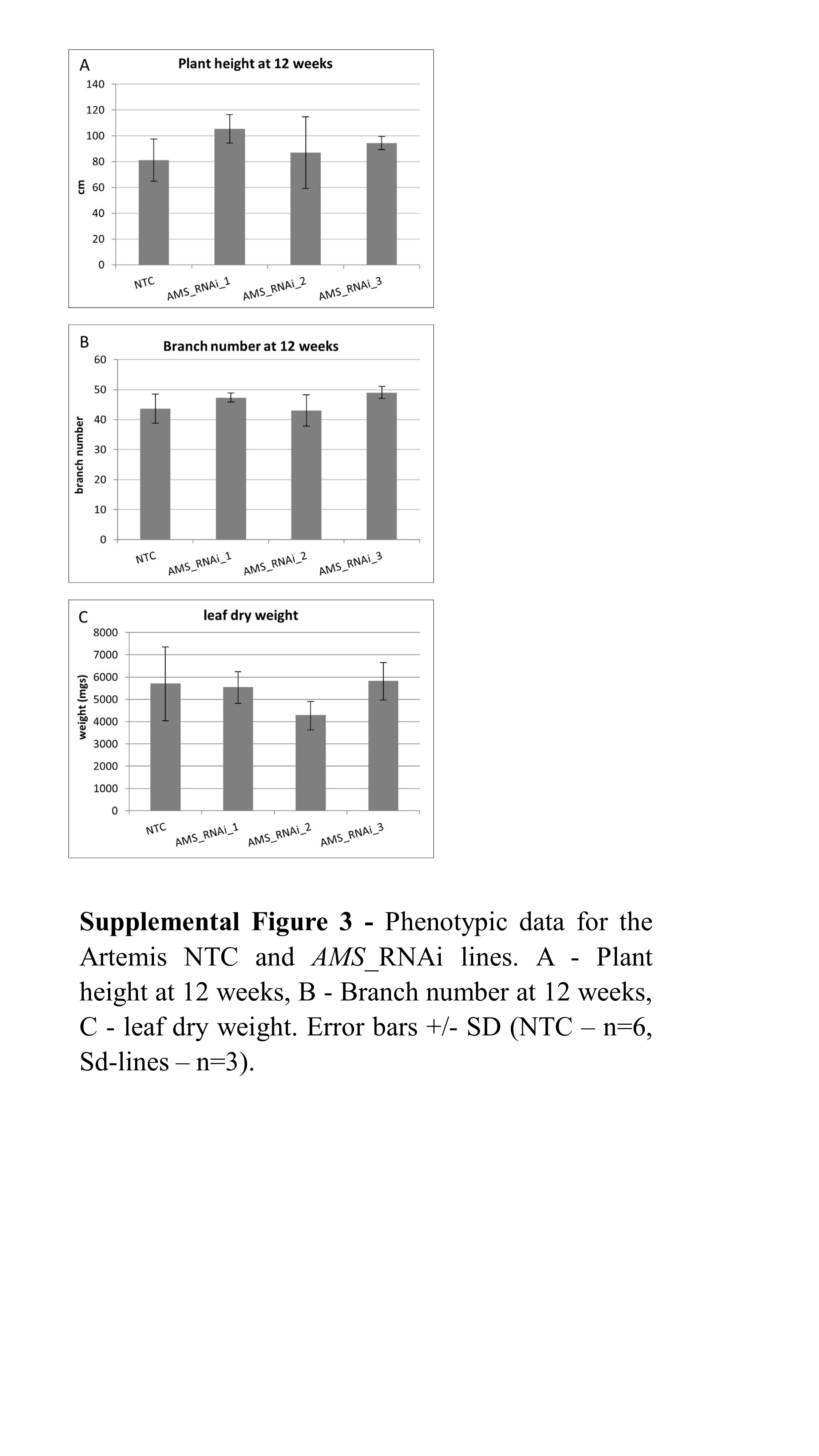

Supplement: Supplementary file 4 [file Image_3.JPEG]
